# Biological Sequence Classification: A Review on Data and General Methods

**DOI:** 10.34133/research.0011

**Published:** 2022-12-19

**Authors:** Chunyan Ao, Shihu Jiao, Yansu Wang, Liang Yu, Quan Zou

**Affiliations:** ^1^ School of Computer Science and Technology, Xidian University, Xi’an, China.; ^2^ Yangtze Delta Region Institute (Quzhou), University of Electronic Science and Technology of China, Quzhou, China.; ^3^ Institute of Fundamental and Frontier Sciences, University of Electronic Science and Technology of China, Chengdu, China.

## Abstract

With the rapid development of biotechnology, the number of biological sequences has grown exponentially. The continuous expansion of biological sequence data promotes the application of machine learning in biological sequences to construct predictive models for mining biological sequence information. There are many branches of biological sequence classification research. In this review, we mainly focus on the function and modification classification of biological sequences based on machine learning. Sequence-based prediction and analysis are the basic tasks to understand the biological functions of DNA, RNA, proteins, and peptides. However, there are hundreds of classification models developed for biological sequences, and the quite varied specific methods seem dizzying at first glance. Here, we aim to establish a long-term support website (http://lab.malab.cn/~acy/BioseqData/home.html), which provides readers with detailed information on the classification method and download links to relevant datasets. We briefly introduce the steps to build an effective model framework for biological sequence data. In addition, a brief introduction to single-cell sequencing data analysis methods and applications in biology is also included. Finally, we discuss the current challenges and future perspectives of biological sequence classification research.

## Introduction

Biological sequences generally refer to sequences of nucleotides or amino acids. Different biological sequences use different experimental methods to obtain sequences. For example, the protein sequence was obtained through experiments such as protein N/C-terminal sequencing, Edman degradation sequencing, complete protein sequence determination, and monoclonal antibody sequencing based on polymerase chain reaction amplification [1]. The nucleotide (RNA and DNA) sequences are obtained using third-generation sequencing technologies such as nanopore sequencing [2–4]. In addition, nanopore single-molecule sequencing technology combined with the dynamic time series data analysis method was used for sequence analysis. Han et al. [5] developed a new algorithm named cwDTW based on dynamic time warping (DTW) to evaluate DNA sequence similarity lifting by cwDTW for the DNA sequence comparison task. In addition, dynamic time series data analysis methods were more widely used in biological sequence analysis: DTW combined with hidden Markov model (HMM) profile was used for protein homolog search [6], and DTW was also used in combination with HMM–HMM for protein fold recognition studies [7] and DTW for subcellular localization classification studies [8] and DNA sequence classification [9]. In the DNA alignment part of the gene signal, DTW was used for position and length alignment of the gene signal [10]. New algorithms have been developed on the basis of DTW, such as the UCR–DTW algorithm applied to DNA sequence data for multiple sequence comparison [11]. Dynamic time series data analysis methods are also applied in several other fields [12–17]. The continuous and rapid development of gene sequencing technology in the post-genome era has produced an exponentially increasing abundance of biological sequence data. With the continuous growth of biological sequence data, researchers hope to comprehensively understand the biological function of the relevant data by mining and analyzing these data with effective computational methods. Therefore, the development of biological sequence data promotes the application of computer technology in its field. Biological sequence classification is a branch of biological sequence analysis research [18,19], which includes many research directions, and the classification of biological sequence functions and modifications is one of them, involving DNA, RNA, and amino acid sequences.

With the fast accumulation of biological sequence data, effective analysis, mining, and visualization of these data have become increasingly difficult tasks. Traditional wet experimental methods such as chromatin immunoprecipitation sequencing, liquid chromatography (LC), mass spectrometry (MS), LC-MS, RNA sequencing (RNA-seq), deoxyribonuclease sequencing, DNA sequencing, chromatin immunoprecipitation, and high-performance LC [20–23] can identify modification sites accurately and reliably. However, they are always prohibitively costly, both in time and financially, thereby limiting the validation and analysis process of biological sequences. The development of machine learning (ML), data mining, and associated technologies in the field of computer science has promoted research in biological sequence data analysis and mining. ML-based methods for biological sequence function prediction/analysis have become popular over the past few years because of their efficiency. Most prediction tasks in the field of biological sequence analysis can be transformed into classification tasks with ML, i.e., binary or multiclass classification tasks. The most widely used traditional ML algorithms in this field include random forest (RF), support vector machine (SVM), naive Bayes (NB), logistic regression (LR), decision tree (DT), light gradient boosting machine (LGBM), and extreme gradient boosting (XGBoost) [19,24,25]. Deep learning has also seen increasing use in biological sequence classification research [26,27].

Various ML-based biological sequence predictive tools have been developed in recent years. Some researchers reviewed and evaluated these biological sequence classification tools. Chen et al. [28,29] reviewed the identification method of RNA modification sites and also discussed the biological function of RNA methylation and its relationship with human diseases. Challenges and developments for future research on RNA modification sites are discussed. However, these reviews only reviewed and discussed the prediction research of RNA modification sites and did not collect relevant data and provide data downloads. In addition, there are some reviews summarizing the computational tools of RNA modification sites for one species or specifically for one or two types of RNA modification sites [30–32]. For the study of protein posttranslational modification (PTM) site prediction, Ramazi et al. [33] and He et al. [34] reviewed online databases relevant to PTM research and a large number of PTM prediction tools. However, the types of PTMs involved in these reviews are not comprehensive, nor do they provide relevant data downloads. Aiming at the classification of imbalanced data, Dou et al. [35] systematically reviewed the prediction research of imbalanced PTM classification and collated the related data on a webpage. It also analyzes the challenges and solutions in unbalanced classification research. Furthermore, some reviews have summarized and analyzed one or more types of modification site computational tools [36,37]. At present, there are few systematic reviews of the classification of biological sequences (DNA, RNA, and amino acids). Focusing on biological sequence modification, we surveyed computational tools for modification of biological sequences and provided downloads of relevant data (http://lab.malab.cn/~acy/PTM_data/), but did not provide a detailed explanation of the performance of each method shown [38].

Building on previous research, we update the biological sequence modification data and demonstrate the performance of each method. In addition, a systematic review of the classification studies of DNA, RNA, and amino acid sequences was carried out. First, the procedure for constructing reliable predictive models for biological sequences is introduced. Second, by comprehensively collecting biological sequence classification data and various computational tools, a webpage with downloadable classification data was constructed, and the dataset, prediction results, and web address were displayed. Finally, we provide suggestions for the challenges of biological sequence classification research and topics for future research. This review is devoted to describing the classification of functions and modifications of biological sequences (DNA, RNA, and amino acids) and analyzes current or popular research by collecting relevant methods and data. It also provides a platform to aggregate and display relevant data and general methods for functional and modification classification research of biological sequences.

## General Scheme and Principles of ML in Biological Sequence Classification

**Fig. 1.**
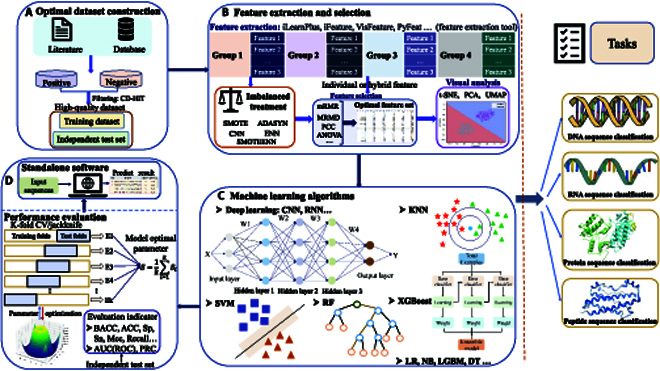
Flowchart for developing machine learning-based predictive models for biological sequences.

Below, we provide the general procedure for constructing reliable ML models of different biological sequences. As shown in Fig. 1, the task flow mainly includes 4 steps: (a) dataset construction, (b) sequence representation and feature selection, (c) model training and evaluation, and (d) web server or standalone software implementation. Each step is briefly elaborated as follows.

### Dataset construction

**Fig. 2.**
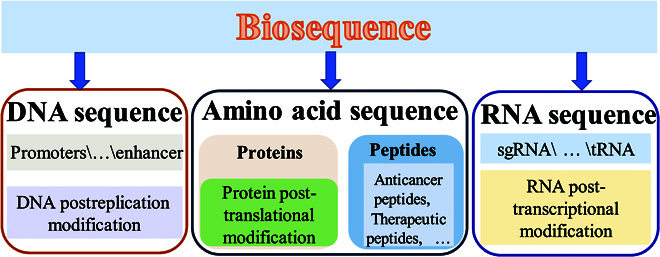
Biological sequence type.

The first task of accurately identifying biological sequences is the collation of a high-quality benchmark dataset. The rough data types contained in the biological sequences are shown in Fig. 2. To construct a biological sequence dataset, the researcher should first collect biological sequence data based on DNA, RNA, and amino acids from related databases and literature. Most of the amino acid sequence data can be obtained from UniProt (https://www.uniprot.org/). The modified sequence data of DNA, RNA, and amino acids has a specially collected database, as shown in Table S1. The initial dataset will have repetitive sequences or sequences with high homology, which will affect the performance of the model. To eliminate these factors, the CD-HIT software [39] could be used to remove the same sequence or a homologous sequence. The threshold range is usually 30% to 90%. The lower the threshold, the lower the homology similarity sequence. The selection of the threshold was determined by the size of the dataset. The obtained dataset could then be divided into a training dataset and an independent test set for the next experiment.

### Sequence representation and feature selection

**Fig. 3.**
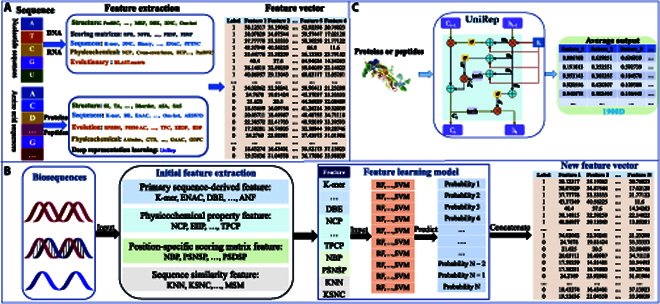
The commonly used feature extraction methods.

Sequence feature extraction is a crucial step in biological sequence classification research. Converting biological sequences into effective mathematical expressions to truly reflect the inherent correlation with the target has an important impact on the performance of the prediction model. The researchers have proposed various feature descriptors for 3 types of biological sequences. Fortunately, several software platforms have been developed to generate these features quickly and easily, including iLearnPlus [40], PyFeat [41], iFeature [42], BioSeq-Analysis 2.0 [43], VisFeature [44], and POSSUM [45]. Some of these open-source platforms have graphical or web-based interfaces that can be easily used even by biologists with no programming background. The biological interpretation of these feature encoding methods has been comprehensively summarized in these studies. There are 7 major encoding schemes for DNA and RNA sequences, including nucleic acid composition, residue composition, position-specific tendencies of trinucleotides, electron–ion interaction pseudopotentials, autocorrelation and cross-covariance, physicochemical property, and pseudo-nucleic acid composition. In terms of proteins and peptides, the feature descriptors could be divided into 8 categories, i.e., amino acid composition, grouped amino acid composition, autocorrelation, quasi-sequence–order, pseudo-amino acid composition, residue composition, physicochemical property, and evolutionary information in the form of a position-specific scoring matrix (PSSM). Overall, these feature extraction methods mainly generate numeric vectors by encoding the composition, biological physical, and biological chemical properties of biological sequences for subsequent ML tasks. On the other hand, Wei et al. [46] proposed a novel feature representation learning scheme that integrates the class and probabilistic information into features, which has been demonstrated to be more informative and effective than the traditional sequence-derived features. Because of its automatic feature extraction and powerful feature representation ability, deep learning has also been widely used for sequence analysis of biological sequences. On the basis of the principle of transfer learning, pretrained deep learning models have emerged to encode sequences in a deep representation learning way. Successful examples of these methods include unified representation (UniRep), Tasks Assessing Protein Embeddings (TAPE), MULocDeep, and BiLSTM embedding model [47–50]. The typical feature extraction methods are shown in Fig. 3.

Most researches on biological sequence classification use multiple feature representation methods or feature fusion methods to represent sequence information. Multiple feature extraction methods or mixed features produce redundant features and result in high-dimensional features. Therefore, it is necessary to apply feature selection methods to reduce dimensionality and improve the performance of the prediction model. The most commonly used feature selection methods include analysis of variance [51], *F*-score [52], maximum relevance and minimum redundancy [53], maximal relevance–maximal distance [54], Pearson correlation coefficient [55], RF, and LGBM algorithms [56]. These techniques are also applied in conjunction with sequential forward feature selection [57] as an effective 2-step feature selection method.

### Model training and evaluation

ML mainly includes 3 types of technologies: unsupervised learning, supervised learning, and semi-supervised learning. Supervised or semi-supervised learning is commonly used in sequence classification tasks. The main goal of ML algorithms is to rigorously train the model to accurately classify any unseen data. Depending on the type of input data, different classification tasks are performed. During model training, the inputs (*X*) are the feature vectors generated by feature extraction or feature selection methods from the training dataset and the response variable (*Y*). The *X* and *Y* of the input model vary according to the data type, for example, for a DNA enhancer classification task, the relevant data are the DNA enhancer sequence, i.e., *X* is an encoded numerical representation in a form suitable for use in ML model (usually is a scheme for categorical) data. In the binary classification study, *Y* is the label 1, and 0 (−1) corresponds to the positive sample and the negative sample, respectively. Similarly, for multicategory, *Y* is the label corresponding to different types of samples. The ML classifier learns the relationship between *X* and *Y*. It makes subsequent predictions for any newly provided datasets. The main objective of a well-performing ML model is to generalize from training data to independent data. In the research on the function and modification classification of biological sequences, we expect to build a predictive model through an optimized ML algorithm to achieve high-accuracy identification of whether an unknown sequence is a certain function or modified sequence.

The commonly used ML algorithms for biological sequence classification are shown in Fig. 1C. Deep learning has been widely used for biological sequence classification in recent years. Greener et al. [58] detailed the application of deep learning in biological modeling and the different models of deep learning, including basic neural networks, convolutional neural networks (CNN), multilayer perceptrons, and recurrent neural networks (RNN). CNN and RNN have been used for the prediction research of biological sequence functions and modification sites. CNN is composed of convolutional, nonlinear, and ensemble layers, and RNN is designed to use the sequential information of the input data to make circular connections between building blocks such as gated recursive units or long short-term memory units. For example, Zou et al. [59] developed a CNN-based classifier Gene2vec for prediction of N6-methyladenosine (m6A) modification sites in mammalian messenger RNA (mRNA) sequences. The classifier CNN contains multiple unit structures such as 2 single-layer convolutional layers, pooling layers, and filtering layers. In this method, the m6A-modified sequence of mRNA is input; the feature vector is obtained by different feature encoding methods, and the feature vector is input into CNN; and the final result is the predicted probability of passing the vote.

In addition, Huang et al. [60] developed an RNN deep learning classifier named BERMP for m6A modification sites in different species, which is based on bidirectional gated recurrent unit. The obtained m6A modification site sequences of different species are encoded by the feature extraction method, the obtained feature vector is input into the RNN classifier, and the corresponding probability is output. Experiments show that the BERMP deep learning framework is more suitable for prediction tasks on large datasets. Compared with traditional ML algorithms, deep learning algorithms have more powerful sequence information extraction capabilities and allow for more accurate biological sequence classification. Commonly used deep learning algorithms are shown in Fig. 1. Traditional ML algorithms can be retrieved from scikit-learn (sklearn) [61]. A variety of deep learning frameworks for training and implementing deep learning models are available, including PyTorch, TensorFlow, and Keras [62]. To summarize, the appropriate classification algorithm is chosen according to the classification task, and the performance of the model can be optimized through parameter selection. Moreover, it is necessary to avoid overfitting and underfitting the model.

The following 3 methods are commonly used to evaluate the performance of constructed models: *k*-fold cross-validation (*k*-fold CV), leave-one-out CV (LOOCV), and independent tests. Both *k*-fold CV and LOOCV are CV techniques. The *k* value of the *k*-fold CV is often 10 or 5. For example, a 10-fold CV means that the dataset is randomly divided into 10 parts, 9 of which are used as the training set, and the remaining 1 is used as the validation test set. All 10 subsets are tested and averaged to obtain the final results of the 10-fold CV. LOOCV is time-consuming to train and is generally only used with smaller datasets. Independent tests can be used to determine model overfitting and evaluate the generalization ability of the model. The following metrics are commonly used in biological sequence prediction to demonstrate the performance of the model: Acc, Sp, Sn, and MCC. Balanced accuracy (BACC) is usually employed to measure the accuracy the overall performance of a model trained on imbalanced datasets. A receiver operating characteristic (ROC) curve was drawn with 1 specificity on the abscissa and sensitivity on the ordinate. The area under the ROC curve (AUC) is also commonly used to evaluate the overall predictive performance. These performance evaluation indicators were calculated on the basis of the confusion matrix presented in Table 1.

**Table 1.** Classification confusion matrix.

Prediction	Actual		
	Negative samples	Positive samples	
Negative samples	TN	FN	$$\begin{array}{c} \mathrm{Sp}=\frac{\mathrm{TN}}{\mathrm{TN}+\mathrm{FP}}*100\%\\ \mathrm{Sn}=\frac{\mathrm{TP}}{\mathrm{TP}+\mathrm{FN}}*100\%\\ \mathrm{Acc}=\frac{\mathrm{TP}+\mathrm{TN}}{\mathrm{TP}+\mathrm{TN}+\mathrm{FP}+\mathrm{FN}}*100\%\\ \text{BACC}=\frac{1}{2}\left(\mathrm{Sp}+\mathrm{Sn}\right)*100\%\\ \mathrm{MCC}=\frac{\mathrm{TP}\times \mathrm{TN}-\mathrm{FP}\times \mathrm{FN}}{\sqrt{\left(\mathrm{TP}+\mathrm{FP}\right)\times \left(\mathrm{TP}+\mathrm{FN}\right)\times \left(\mathrm{TN}+\mathrm{FP}\right)\times \left(\mathrm{TN}+\mathrm{FN}\right)}} \end{array}$$
Positive samples	FP	TP	

TP, true positive; TN, true negative; FP, false positive; FN, false negative; Sp, specificity; Sn, sensitivity; Acc, accuracy; BACC, balanced accuracy (for imbalance data); MCC, Matthew’s correlation coefficient.

### Web server development

The developed prediction models and biological sequence datasets should be disclosed to researchers, and a user-friendly web server is highly recommended to facilitate access to prediction tasks and datasets. With the web server, researchers can perform preliminary screening before experimental validation. The web server can be implemented using conventional technologies such as Django, Dash, Flask, Hyper Text Markup Language (HTML), Cascading Style Sheets (CSS), and hypertext preprocessing (PHP). On the other hand, standalone software or source code can benefit the research community if a web server is not provided.

## Progress in Biological Sequence Classification

**Fig. 4.**
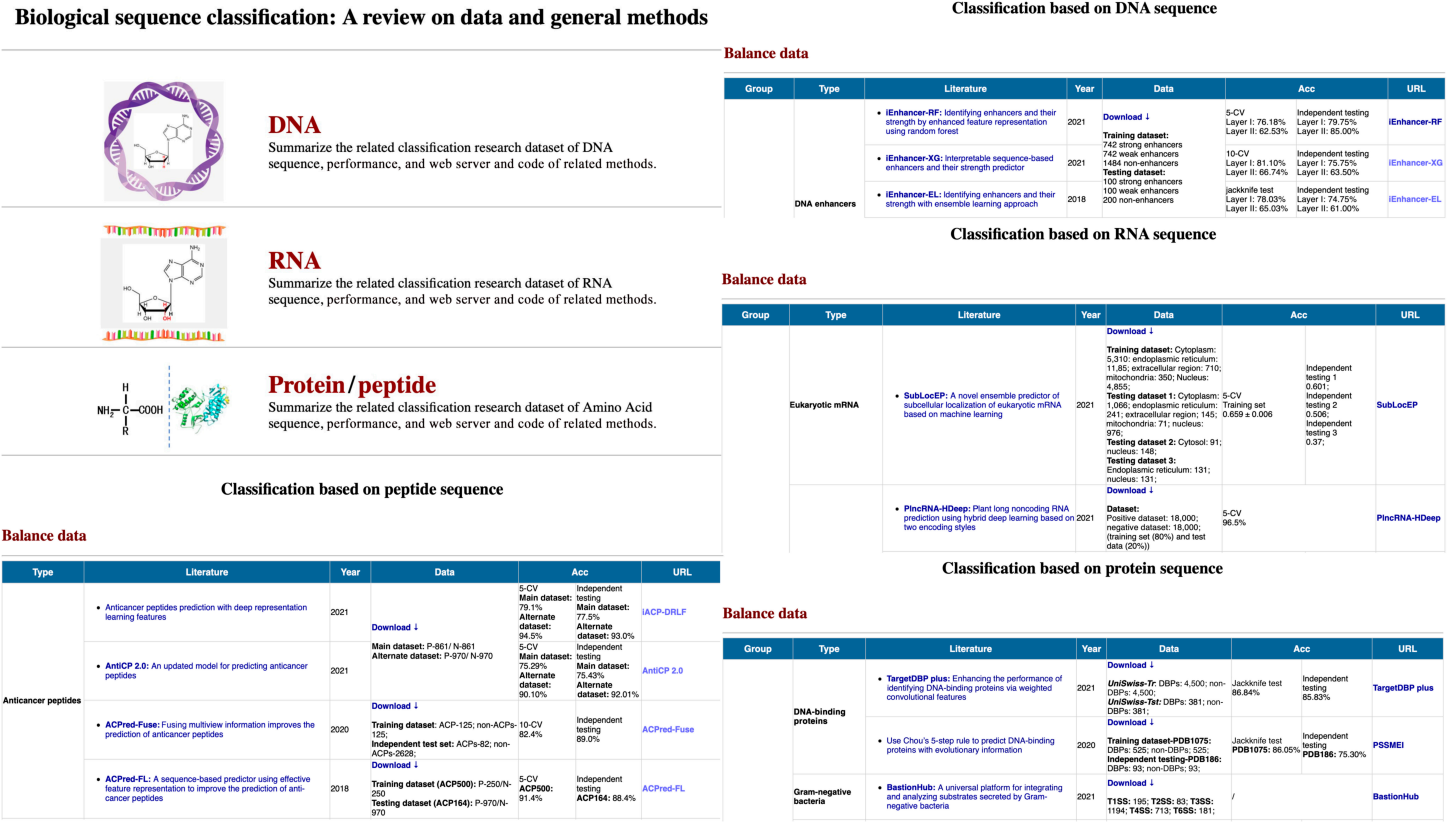
Webpage layout of methods and data related to biological sequence classification (http://lab.malab.cn/~acy/BioseqData/home.html).

Thousands of computational methods have been developed for the prediction of biological sequences. The specific methods are completely different and may seem confusing at first glance. Here, we do not intend to provide a comprehensive literature review of articles of specific prediction tasks or to describe the detailed mathematics behind each ML algorithm. Instead, we focused on the summary of benchmark datasets and model performance for particular biological sequence data. On the basis of this goal, in this review, we provide a comprehensive survey regarding the state-of-art computational methods according to 3 types of biological sequence data (DNA, RNA, and amino acid). We present a wide scope of aspects, including the dataset information, the core algorithms chosen for each method, performance evaluation strategy, and software availability (Tables S2–S4). With these data, we also constructed a long-term support online webpage (*http://lab.malab.cn/~acy/BioseqData/home.html*). This webpage will keep relevant researchers clearly informed of the latest progress on each task. The intricacy of biological sequence data presents pitfalls and opportunities for biological sequence analysis using ML techniques. This webpage provides researchers with the ability to compare the classification methods and download related data quickly and conveniently. As shown in Fig. 4, the webpage summarizes the classification methods and related data of DNA, RNA, protein, and peptide biological sequences, respectively. We have collected hundreds of the most advanced classification tools. According to the number of positive and negative samples in a dataset, we have categorized the classification methods into a balance data table and an imbalance data table. In the tables, we provide the group (unequal-length sequence and equal-length sequence), type, literature name, year, data, prediction result, and web address. Biological sequence datasets can also be directly accessed on the webpage. Our systematic summary of the general methods and data of biological sequence classification will aid the development of biological sequence classification research and alleviate a researcher’s overall time investment. We aim to continuously update the webpage based on the latest research.

### DNA sequences

DNA is a biological macromolecule in living organisms. It carries the genetic information of life and guides the development of biological functions and the exertion of life functions [63]. The research on the function and modification site classification of DNA sequences is conducive to deeply understanding the biological function of DNA sequences. Classification methods developed on the basis of ML have grown with the proliferation of DNA sequence data [64–77]. DNA sequence-based classification methods and related data are shown in Table S2 and are divided into 2 categories according to sequence length: general prediction methods (unequal-length sequences) and modification site prediction methods (equal-length sequences). Unequal-length sequences include DNA enhancers, promoters, nucleosomes, and functional of DNA sequences. Equal-length sequences (DNA modification sites) contain N6-methyladenine (6mA), N4-methylcytosine (4mC), and 5-methylcytosine (5mC).

Here, we mainly focus on several types of DNA sequence classification studies: enhancers, promoters, and functional effects of DNA sequences. For example, a recently developed 2-layer predictor, iEnhancer-XG [64] integrates a variety of sequence features and uses integrated learning to classify DNA enhancers. iEnhancer-XG uses 5 feature extraction methods, including PSSM, *k*-spectrum profile, mismatch *k*-tuple, PseDNC, and subsequence profile, and combines 5 separate output feature vector input integrated learning (Fig. 3B), using XGBoost as a classifier. The training dataset of this method contains 742 strong enhancers, 742 weak enhancers, and 1,484 non-enhancers. An independent test set (100 strong enhancers, 100 weak enhancers, and 200 non-enhancers) was constructed to verify the performance of the model. Under the 10-fold cross-verification and independent test set, the Acc values obtained by the 1-layer predictor were 81.10% and 75.75%, and those obtained by the second layer classifier were 66.74% and 63.50%. However, this performance evaluation should be conducted with a larger dataset, which would likely improve accuracy. Lyu et al. [65] developed a novel method (iPro2L-PSTKNC) for the prediction of promoters in the *Escherichia coli* genome. The predictor adopts the new feature extraction method PSTKNC to the encoding sequence and the obtained feature vector input integration classifier SVM. The number of positive samples and negative samples in the dataset used by this method is 2,860 and 2,860, respectively. The positive sample contains 6 types of promoters: σ^24^-484, σ^28^-134, σ^32^-291, σ^38^-163, σ^54^-94, and σ^70^-1694. The Acc and MCC values obtained by this classification method were 90.05% and 80.13%, respectively. This prediction method uses synthetic minority oversampling technique (SMOTE) to handle data imbalance. For multitype classification with a smaller sample size, the data quantity of the training model can be increased for stronger migration of the model. A novel classification method, DeepATT [66], has been developed on the basis of hybrid deep neural networks and is used for the functional classification of DNA sequences. The data used in this study include a training dataset of 440,000, a validation dataset of 8,000, and an independent test dataset of 450,024. A hybrid neural network, on the basis of deep learning, was used to construct the predictor. The average area under the ROC obtained by DeepATT is 0.94519, and the performance of this method is much higher than other classification methods. This method effectively reduces the number of parameters to ensure classification accuracy and improves the performance of the model.

DNA modification site prediction studies mainly focus on 4mC and 6mA, with relatively few studies on 5mC. Many 4mC modification site prediction tools have been developed on the basis of ML. 4mC-w2vec [67] and Deep4mcPred [69] use deep learning to classify 4mC in multiple species with high accuracy. 4mC-w2vec is a predictor developed for 2 species, *F. vesca* and *R. chinensis*, using training datasets (*F. vesca*: P-3457/N-3457 and *R. chinensis*: P-1938/N-1938) to construct model and an independent test set (*F. vesca*: P-864/N-864, 4320, and 12960 and *R. chinensis*: P-483/N-2415 and 7245) to test the model. In addition, 4mC-w2vec is also used to conduct comparative analyses on data imbalance using deep learning. The accuracies obtained for *F. vesca* and *R. chinensis* were 86.97% and 85.41%, respectively. The accuracy of the independent test set under different proportions was 84.00% to 86.32%. Similarly, Deep4mcPred developed a predictor for 3 species. The training dataset contains *Arabidopsis thaliana* (20,000/20,000), *Caenorhabditis elegans* (20,000/20,000), and *Drosophila melanogaster* (20,000/20,000). The accuracies obtained for *A. thaliana*, *C. elegans*, and *D. melanogaster* were 84.4%, 89.3%, and 87.1%, respectively. Predictors such as EC4mC-SVM [68], 4mcPred-IFL [70], 4mcPred-SVM [78], and 4mCPred [71] use different traditional ML methods to classify 4mC-modified sites. EC4mC-SVM is a predictor developed for *E. coli*. The training dataset was P-388/N-388, and the independent test set was P-134/N-134. The iEC4mC-SVM predictor had a 10-fold CV accuracy of 85.4% and an independent testing accuracy of 83.2%. 4mcPred-IFL [70], 4mcPred-SVM [78], and 4mCPred [71] use the same dataset. These 3 predictors can identify 4mC modification sites in 6 species, namely, *C. elegans*: P-1554/N-1554, *D. melanogaster*: P-1769/N-1769, *A. thaliana:* P-1978/N-1978, *E. coli*: P-388/N-388, *Geoalkalibacter subterraneus*: P-905/N-905, and *Geobacter pickeringii*: P-569/N-569. Through continuous improvement of feature extraction methods and classification algorithms, model accuracy is gradually improved. The Acc value of each species under the CV of 4mcPred-IFL and 4mcPred-SVM is as follows: 4mcPred-IFL (*C. elegans*: 88.00%; *D. melanogaster:* 87.40%; *A. thaliana:* 82.50%; *E. coli*: 89.40%; *G. subterraneus*: 88.60%; and *G. pickeringii*: 90.70%.) and 4mcPred-SVM (*C. elegans*: 81.50%; *D. melanogaster*: 83.00%; *A. thaliana*: 78.70%; *E. coli*: 83.30%; *G. subterraneus*: 83.70%; and *G. pickeringii*: 86.00%).The 4mCPred predictor jackknife CV accuracy and independent test set accuracy are as follows: jackknife test (*C. elegans*: 87.71%; *D. melanogaster*: 87.79%; *A. thaliana*: 83.37%; *E. coli*: 94.97%; *G. subterraneus:* 91.04%; and *G. pickeringii*: 90.89%) and independent testing (*C. elegans*: 82.21%; *D. melanogaster*: 82.63%; *A. thaliana*: 76.52%; *E. coli:* 82.69%; *G. subterraneus*: 83.33%; and *G. pickeringii:* 77.63%). Classification methods for the 6mA modification site sequence include iRicem6A-CNN [73] and iIM-CNN [79], which are both based on CNN in deep learning. 6mA-RicePred [74] and csDMA [75] are based on prediction models constructed using SVM and Extra Trees, respectively. iIM-CNN can identify 6mA modification sites in multiple species. The training datasets are cross-species: P-2768/N-2716, rice: P-880/N-880, and *Mus musculus*: P-1934/N-1934. The accuracy of this method under 5-fold CV is cross-species: 82.40%, rice: 87.50%, *M. musculus*: 96.90%. Similarly, the csDMA predictor uses the same *M. musculus* (1,934/1,934) and rice (880/880) data. In addition, the cross-species data in the training dataset were 2,768/2,716, and the cross-species independent test set was 554/554. The accuracy under 5-fold CV and independent test set is as follows: 5-fold CV (cross-species: 79.90%; rice: 86.10%; and *M. musculus*: 96.60%) and independent testing (cross-species: 81.30%). In addition, iRicem6A-CNN (training dataset: P-154000/N-154000 and independent test set: P-880/N-880) and 6mA-RicePred (training dataset: P-880/N-880 and independent test set: P-154000/N-154000) use the same dataset to classify 6mA modification sites in a rice genome sequence. The accuracy obtained by iRicem6A-CNN (Acc value of 93.82% for 5-fold CV and 96.19% for independent testing) is better than that of 6mA-RicePred (Acc value of 87.27% for 10-fold CV and 85.65% for independent testing). Deep learning shows better performance with large datasets. For the classification study of 5mC modification sites, iPromoter-5mC was used to collect the first large-scale 5mC dataset for classification research. The training dataset was p-55800/N-13950, and the independent test set was P-658861/N-164751. Using the down-sampling method for data imbalance processing, the Acc values obtained from the training dataset and independent test set were 90.16% and 90.22%, respectively.

### RNA sequences

RNA is a biological macromolecule similar to DNA. Unlike DNA, RNA is single-stranded. RNA participates in life activities and performs specific biological functions in cells. Therefore, the classification study of RNA sequences is helpful for the further study of the biological function and modification mechanism of RNA. With improved data mining efficiency for RNA sequence types and unknown modification site sequence data, the development of general classification methods based on ML is also increasing [24,31,80–94]. General methods and related data for RNA sequence classification are summarized in Table S3. RNA sequence classification is divided into 2 categories: (a) unequal length sequences (used for classification of the eukaryotic mRNA, noncoding RNA, pre-microRNA, circular RNA (circRNA), transfer RNA (tRNA), Piwi-interacting RNA, long noncoding RNA (lncRNA), and single guide RNA) and (b) equal length sequences (used for the classification of RNA posttranscriptional modification sites m6A, N1-methyladenosine (m1A), pseudouridine (Ψ), adenosine-to-inosine (A-to-I), 5-methylcytosine (m5C), N2-methylguanosine (m2G), 2′-*O*-methylation (Nm), 5-hydroxymethylcytosine (5hmC), dihydrouridine (D), N6,2′-*O*-dimethyladenosine (m6Am), N7-methylguanosine (m7G), 5-methyluridine (m5U), and inosine (I)).

For the classification research of RNA sequences of unequal length, we introduce the following: SubLocEP [80] is a predictor developed with ML for the subcellular localization of eukaryotic mRNA. This method adopts integrated ML to classify the cytoplasm, endoplasmic reticulum, extracellular region, mitochondria, and nucleus. The training dataset includes cytoplasm: 5,310; endoplasmic reticulum: 1,185; extracellular region: 710; mitochondria: 350; and nucleus: 4,855. The independent test set contains 3 datasets, namely, D1 (cytoplasm: 1,066; endoplasmic reticulum: 241; extracellular region: 145; mitochondria: 71; and nucleus: 976), D2 (cytosol: 91 and nucleus: 148), and D3 (endoplasmic reticulum: 131 and nucleus: 131). The accuracy using this method was as low as 0.659 ± 0.006. The accuracies of the 3 independent testing sets were also low: 0.601, 0.506, and 0.37. The accuracy of this method requires further improvement. A classification tool, CirRNAP [81], was developed for circRNA sequences. This method performs 3 categories of classification work: circRNA vs. protein coding gene (PCG) (training dataset: circRNA-10000/PCG-8000 and testing dataset: circRNA-4084/PCG-1533), circRNA vs. lncRNA (training dataset: circRNA-10000/lncRNA-10000 and testing dataset: circRNA-4084/lncRNA-9722), and circRNA vs. non-circRNA (training dataset: circRNA-1800/non-circRNA-1800 and testing dataset: circRNA-282/non-circRNA-282). Under 10-fold CV, the accuracy of the 3 categories of classification work was 81.50% (circRNA vs. PCG), 80.20% (circRNA vs. lncRNA), and 78.20% (stem cell vs. not), respectively. The accuracy under the independent test set was 82.70% (circRNA vs. PCG), 85.40% (circRNA vs. lncRNA), and 81.20% (stem cell vs. not), respectively. The training dataset was relatively large, and the classification model had strong generalization ability. tRNA-Predict [82] is based on an ensemble classifier. The training dataset contains positive samples of 623 and negative samples of 1,183. The tRNA-Predict predictor had a 10-fold CV accuracy of 95.10%. Research on tRNA classification occurred relatively early, although data are limited. On the basis of this method, a more stable model can be constructed with larger datasets. In response to the previously predicted insufficient activity of sgRNA on the target gene, Niu *et al.* [85] developed SgRNA-RF, which uses 5 datasets, some of which are severely imbalanced between positive and negative samples. For example, the correlation data of the G5 sequence, hek293t: training dataset: P-1615/N-428 and testing dataset: P-404/N-108; hct116: training dataset: P-3090/N-428 and testing dataset: P-783/N-108; hela: training dataset: P-5923/N-428 and testing dataset: P-782/N-108; and h160: training dataset: P-1973/N-428 and testing dataset: P-494/N-108. The positive and negative sample ratios were between 3.77 and 13.84. To deal with the imbalance problem, the CS-SMOTE method was used to improve the BACC. The Acc value under 10-fold CV was G17-84.7%, Gr-69.4%, and G5-(hct116: 96.9%, hek293t: 94.0%, hela: 97.74%, and h160: 94.10%). The Acc value of the independent test set was G17-86.3%, Gr-91.6%, Gnr-89.4%, Gm-93.8%, and G5-(hct116: 96.50%, hek293t: 78.70%, hela: 97.90%, and h160: 97.30%).

There are only about dozen types involved in the prediction research of RNA posttranscriptional modification sites. On the basis of ML and RNA modification data, many classification methods have been developed (Table S3). Among them, many predictors have been developed specifically for m6A modification sites. Data and species of m6A modification sites are the most common. For example, the m6A prediction tools DNN-m6A [90] and iRNA-m6A [91], developed on the basis of deep learning, used the same dataset and analyzed the brain, kidney, and liver of human, mouse, and rat, respectively. In mouse, m6A modification sequence data were also collected from the heart tissue. The training dataset was human (brain: 4,605/4,605; kidney: 4,574/4,574; and liver: 2,634/2,634), mouse (brain: 8,025/8,025; heart: 2,201/2,201; kidney: 3,953/3,953; liver: 4,133/4,133; and testis: 4,704/4,704), and rat (brain: 2,352/2,352; kidney: 3,433/3,433; and liver: 1,762/1,762). The independent test set was human (brain: 4,604/4,604; kidney: 4,573/4,573; and liver: 2,634/2,634), mouse (brain: 8,025/8,025; heart: 2,200/2,200; kidney: 3,952/3,952; liver: 4,133/4,133; and testis: 4,706/4,706), and rat (brain: 2,351/2,351; kidney: 3,432/3,432; and liver: 1,762/1,762). The accuracy values obtained by DNN-m6A and iRNA-m6A were 72.95% to 83.38% and 68.79% to 81.78%, respectively. HSM6AP [92] was developed for *Homo sapiens* using data originally from WHISTLE [95], which includes 6 base–resolution datasets (from 5 cell types). Using these data, the authors reconstructed training and independent test datasets. Experimental results show that HSM6AP outperforms previous prediction tools, with average AUC values of approximately 0.975 and 0.900 on full transcript and mature mRNA data, respectively. iMethyl-Deep [93], DeepM6APred [94], and RAM-ESVM [96] also used the same training dataset (D1: 1,307/1,307) to train the model. After continuous improvement of the method, the Acc value was increased to 89.19%, 80.50%, and 78.35%, respectively. For other types of modification sequence data, most studies improve classification methods using existing datasets or develop new classification methods by adding data. For Ψ modification sites, RF-PseU [87] and iPseU-CNN [97] were trained and tested on the same dataset. The training dataset was *H. sapiens* (495/495), *Saccharomyces cerevisiae* (314/314), and *M. musculus* (472/472). The independent test set was *H. sapiens* (100/100) and *S. cerevisiae* (100/100). The accuracy obtained by RF-PseU and iPseU-CNN was 64.30% to 77.00% and 66.68% to 73.50%, respectively. In comparison, Dou’s method [88] increased the number of training dataset (*H. sapiens:* 495/495; *S. cerevisiae:* 319/319; and *M. musculus:* 495/495) sequences of *S. cerevisiae* and *M. musculus*. For the 3 species, the 10-fold CV accuracy obtained was 62.73%, 70.54%, and 71.72%, respectively. At the same time, the sample size of *S. cerevisiae* was increased in the independent test set. The accuracy was 60.20% and 77.00%, respectively. In addition, for m2G modification sites, a predictor RFhy-m2G was developed on the basis of RF. The training dataset contained *H. sapiens* (41/541), *M. musculus* (27/427), and *S. cerevisiae* (60/283). The independent test set was *H. sapiens* (5/60), *M. musculus* (3/47), and *S. cerevisiae* (7/31). Because of data imbalance, the RFhy-m2G [24] classification method can correct for the imbalance of positive and negative samples using SMOTE. The accuracy of the 10-fold CV for the 3 species was 99.82%, 100%, and 99.65%, respectively. The accuracy of the independent test set was 94.17%, 90.43%, and 100%, respectively. Dou’s method [98] (training dataset: 140/298 and independent test set: 36/76) and iRNA-m5C_NB [99] (training dataset: 127/808 and independent test set: 157/1,000) both use the hybrid sampling method SMOTE and undersampling method edited nearest neighbors (ENN) (SMOTEENN) to deal with data imbalance. For Dou’s method, the accuracy of the training dataset and the independent test set was 97.24% and 93.75%, respectively. For iRNA-m5C_NB, the accuracy of the training dataset and the independent test set was 82.20% and 74.85%, respectively. For multitype RNA modification site prediction, iMRM [31] is a predictor developed on the basis of ML for multitype RNA modification classification. The species involved include *H. sapiens* (m1A: 6,366/6,366; m5C: 120/120; m6A: 1,130/1,130; Ψ: 495/195; and A-to-I: 3,000/3,000), *S. cerevisiae* (m1A: 483/483; m5C: 211/211; m6A: 1,307/1,307; and Ψ: 313/314), and *M. musculus* (m1A: 1,064/1,064; m5C: 97/97; m6A: 725/725; and Ψ: 472/472). The lowest accuracy obtained by the iMRM method was 66.47%, and the highest accuracy was 100%. This method has room for improvement in the accuracy of Ψ modification. Another predictor, MultiRM [86], was developed on the basis of deep learning and covers 12 types of RNA modification (m6A: 65,178; Ψ: 3,137; m1A: 16,380; m6Am: 2,447; Am: 1,591; Cm: 1,878; Gm: 1,471; Um: 2,253; m5C: 12,936; m7G: 1,036; m5U: 1,696; and I: 52,618). The MultiRM predictor performs a multiclassification task, and the accuracy obtained was 65.00% to 92.00%. In order to solve the problem of data imbalance, the MultiRM method introduces online hard examples mining and uncertain weights to improve accuracy. For other RNA posttranscriptional modifications, information on the related classification methods of Nm, m2G, m7G, 5hmC, and D is shown in Table S2.

### Protein sequences

Proteins are composed of 20 different types of amino acids, which are present in nature and encoded by DNA sequences. Proteins play important roles in the cells of organisms, including catalysis of reactions, recognition, regulation, cell signaling, membrane transport, and the provision of structure [100]. As the gap between the number of proteins being discovered and their functional characterization grows (especially due to experimental limitations), reliable prediction of protein function by computational means has become crucial [101–111]. Common protein classification tasks include conventional unequal-length and equal-length sequences (Table S4). By collecting proteins with many studies and significant significance, the conventional unequal length sequence types including DNA-binding proteins, RNA-binding proteins (RBPs), secretory proteins, cancerlectin, phage virion protein, cell wall lytic enzymes, thermophilic proteins, major histocompatibility complex, antioxidant proteins, biological luminescent proteins, electron transport proteins, plant pentatricopeptide repeat, sub-Golgi protein, and type III fluctuation systems. The classification of protein equal-length sequences is related to the prediction of protein PTM sites. Protein PTM involves the modification or addition of chemical groups that occur on amino acid residues. A review of protein PTMs showed that there were about a dozen types of modifications, which have been shown on online webpages.

DNA-binding proteins and RBPs are special binding proteins that are formed by the combination of nucleotides and proteins. The following 3 methods were applied to a classification study of RBPs: rBPDL [106], RBPro-RF [107], and TriPepSVM [108]. The dataset used by rBPDL contained RBP sequences (72,226) and negative sequences (137,003). Deep learning was used for prediction research. The AUC was higher than 0.932. The positive and negative samples in the dataset used by the RBPro-RF and TriPepSVM methods were imbalanced. The ratio of positive and negative samples in the RBPro-RF training dataset was 2.55. The author adopted SMOTE for imbalanced processing. The training dataset was P-2780/N-7093. The constructed independent testing set contained 3 species: *H. sapiens* (RBPs: 967 and non-RBPs: 597), *S. cerevisiae* (RBPs: 354 and non-RBPs: 135), and *A. thaliana* (RBPs: 456 and non-RBPs: 37). Under 10-fold CV, the Acc value of the model was 97.43%. The Acc values obtained from the performance of the 3 species’ test models were 95.63%, 88.82%, and 92.35%, respectively. The training dataset constructed by the TriPepSVM predictor was human (1,625/10,834), *Salmonella* (275/1,273), and *E. coli* (460/3,404). The independent test set included human (181/1,204), *Salmonella* (31/142), and *E. coli* (52/379). TargetDBP plus is a newly developed DNA-binding protein classification tool [101], which uses weighted convolution features to encode sequence information. This method used 4,500 positive and negative samples in the training dataset, and the positive and negative samples of the independent test set were 381. The accuracies of the jackknife test and independent test were 86.84% and 85.83%, respectively. TargetDBP plus adds the training dataset and an independent test set based on the PSSMEI [102] method and improves the accuracy of the model by improving the method. Moreover, some classification tasks have fewer sequences of data. The quality of the dataset determines the prediction model. For cell wall lytic enzyme classification research based on a single dataset (lyases: 68 and non-lyases: 307), 3 classification tools have been developed: CWLy-RF [103], CWLy-SVM [104], and Jing’s method [105]. The accuracy obtained was 96.09%, 95.50%, and 99.19%, respectively. Although the accuracy obtained was as high as 99.19%, the dataset was too small, and the migration performance of the model was poor.

The classification of protein equal-length sequences is related to the prediction of protein PTM sites (Table S4). Protein PTM involves the modification or addition of chemical groups that occur on amino acid residues. A review of protein PTMs showed that there were about a dozen types of modifications, which have been shown on online webpages (http://lab.malab.cn/~acy/BioseqData/Protein.html). According to the related database of protein PTMs, the top 3 types of modification data are phosphorylation, acetylation, and ubiquitination. Phosphorylation predictors developed on the basis of deep learning are DeepPPSite [112] and DeepPSP [113]. DeepPPSite constructed a training dataset (S: 4,316/4,316; T: 1,551/1,551; and Y: 553/553) and an independent test set (S: 2,773/17,118; T: 941/6,258; and Y: 210/1,296). The accuracy of the 10-fold CV was 80.38% (S), 80.01% (T), and 77.73% (Y). The accuracy of the independent test set was 78.91% (S), 84.81% (T), and 82.73% (Y). The accuracy of the independent test set was 78.91% (S), 84.81% (T), and 82.73% (Y). The datasets used by the DeepPSP method were a training dataset (S/T: P-165787/N-879507 and Y: P-28965/N-134997) and a testing dataset (S/T: P-18588/N-102113 and Y: P-3248/N-14504). For more than 4 positive and negative samples, the developers of DeepPSP used a deep learning algorithm to process the data and solve the imbalance problem. iPhoPred [114] and PhosPred-RF [115] were based on predictors developed by SVM and RF, respectively. The dataset used by iPhoPred was small, with a SerD of 600 and TyrD and ThrD of 200. The AUCs obtained were 0.904, 0.992, and 0.990, respectively, and the model may have been overfitted. PhosPred-RF uses the training dataset (S-type: 4,316/4,316; T-type: 1,551/1,551; and Y-type: 553/553) to train the model, using an independent test set (S-type: 2,273/17,618; T-type: 941/6,258; and Y-type: 296/1,210) to test model performance. The AUCs of the 10-fold CV were 0.851, 0.818, and 0.761, respectively. The AUCs of the independent test set were 0.715, 0.683, and 0.654, respectively. Acetylation is the second most modified type of protein PTM. Since its discovery, many classification methods have been developed on the basis of ML. For lysine acetylation modification sites, DNNAce [111] and ProAcePred [116] used the same positive sample sequence data related to 9 species (training dataset: *Archaea*: 193/193,1590; *Bacillus subtilis*:1040/1040,5772; *Corynebacterium glutamicum*: 1021/1021,4333; *Erwinia amylovora*: 95/95,718; *E. coli*:1919/1919,1919; *Geobacillus kaustophilus*:189/189,1025; *Mycobacterium tuberculosis*: 866/866,3926; *Salmonella typhimuricum*: 174/174,1467; and *Vibrio parahemolvticus*: 1065/1065,5938). DNNAce and ProAcePred use independent test set 1 (*Archaea*: 21/21; *B. subtilis*: 115/115; *C. glutamicum*: 113/113; *E .amylovora*: 10/10; *E. coli*:213/213; *G. kaustophilus*:21/21; *M. tuberculosis*:96/96; *S. typhimuricum*:19/19; and *V. parahemolvticus*:118/118) and independent test set 2 (*Archaea*: 21/176; *B. subtilis*:115/641; *C. glutamicum*: 113/481; *E. amylovora*: 10/80; *E. coli*: 213/213; *G. kaustophilus*: 21/114; *M. tuberculosis*: 96/436; and *S. typhimuricum*: 19/163), respectively. The accuracy obtained by DNNAce and ProAcePred was 63.08% to 98.26% and 69.00% to 98.30%, respectively. Another predictor, DeepAcet [52] was developed on the basis of deep learning. This method uses a relatively large dataset (training dataset: P-12886/N-12886 and independent testing dataset: P-3221/N-3221). Under the 10-fold CV and independent test sets, the accuracy obtained was 84.95% and 84.87%, respectively. Less data are available for ubiquitination compared to the other 2 modification types. In the latest report, the predictor DeepTL-Ubi [117] covered 8 species, including the *H. sapiens* (31,162/31,162), *M. musculus* (7,746/7,748), *S. cerevisiae* (3,506/3,506), *Rattus norvegicus* (1,226/1,226), *Aspergillus nidulans* (2,299/2,299), *A. thaliana* (586/587), *Toxoplasma gondii* (424/424), and *Oryza sativa* (308/308). The training set using this method showed a low accuracy (0.500–0.604). The accuracy for different species needs to be improved. For *A. thaliana*, the predictor CNNAthUbi [118] was developed on the basis of a deep learning CNN. The training dataset consists of positive samples (2,043) and negative samples (6,130), and the independent test set consists of positive samples (511) and negative samples (1,533). The Acc value for the 10-fold CV and independent test set was 85.38% and 85.36%, respectively. In addition, for the general methods and data of protein PTM imbalance classification, Dou et al. [35] developed ImbClassi_PTMs, which comprehensively summarized the performance and related data of the general methods, provided online services for dataset downloads, and introduced general methods.

### Peptide sequences

Peptides are an important class of naturally occurring biologics, which are amino acid sequences with a length of less than 50. Their unique structures provide them with various functions as hormones, biological messengers, growth factors, anti-infectives, and neurotransmitters [119]. Accurate prediction of biological peptides plays an important role in the discovery and development of efficient peptide-based drugs. However, experimental methods available for the discovery and synthesis of biological peptides are costly, time-consuming, and labor-intensive. Furthermore, this characteristic makes it especially difficult to develop well-performing predictive models by ML due to the short sequences, the more difficult it becomes to extract statistical information. The most commonly studied peptides in classification research include anticancer peptides, antihypertensive peptides, antitubercular peptides, therapeutic peptides, toxic peptides, cell-penetrating peptides, hemolytic peptides, and bitter peptides (Table S4).

There are dozens of classification methods developed for these peptide sequences [120–125], among which, there are many studies of anticancer peptides, therapeutic peptides, and cell-penetrating peptides that are specifically aimed at disease treatment. iACP-DRLF [120] and AntiCP 2.0 [121] are anticancer peptide prediction tools developed by ML. Both methods used the same dataset. The 2 datasets, consisting of both the training dataset and the independent test set, were the main dataset (861/861) and the alternate dataset (970/970). Compared with AntiCP 2.0, iACP-DRLF has improved accuracy in both the training (main dataset: 79.10% and alternate dataset: 94.5%) and independent test sets (main dataset: 77.50% and alternate dataset: 93.0%). However, the accuracy for the main dataset is less than 80%. The ACPred-Fuse [122] (training dataset: 125/125 and independent test set: 82/2628) and ACPred-FL [126] (training dataset: 250/250 and independent test set: 970/970) classification methods used different datasets. The accuracy of 5-fold CV was 82.4% and 91.4%, respectively, and the accuracies of the independent test set were 89.0% and 88.4%, respectively. The training dataset of this method was relatively small (ACPred-Fuse: ACPs-125, non-ACPs-125; ACPred-FL (ACP500): P-250/N-250). For the classification study of therapeutic peptides, the classification methods and related data from the past 3 years have been summarized. The ITP-Pred [123] method is developed on the basis of hybrid low-dimensional features and deep learning and is mainly used for the prediction of quorum-sensing peptide (training dataset: 400/400 and independent test set: 40/40) and cell-penetrating peptide (CPP) (training dataset: 740/740 and independent test set: 92/92) peptides. The dataset increased the number of sequences based on previous research. For QSP, the accuracy of 5-fold CV and independent test sets was 87.00% and 87.30%, respectively. Similarly, for CPP, the accuracy of 5-fold CV and independent test sets was 95.10% and 97.50%, respectively. Several classification methods were developed on the basis of the cell-penetrating peptide sequence, and the training dataset used was CPP924 (CPPs-462 and non-CPPs-462). The accuracies obtained by the classification method were all above 90%. However, the accuracy of the CPPred-RF [127] method on the CPPsites dataset was only 71.1%. Similarly, the accuracy obtained by StackCPPred [125] was 78.3%. The TargetCPP [124] method adopts an independent test set to verify the performance of the model. For the CPPind (CPPs: 111 and non-CPPs: 34) dataset, the accuracy obtained was 88.28%. Research data and methods for other types of peptides are constantly being developed and improved.

## Single-Cell Sequencing Data Analysis for Biological Applications

For the analysis of single-cell sequencing data, different analysis processes are used for different sequencing types and research purposes. Single-cell sequencing mainly includes single-cell DNA sequencing, single-cell RNA-seq (scRNA-seq), and single-cell epigenome sequencing, and for single-cell DNA sequencing and single-cell epigenome sequencing, the data analysis process is similar to the traditional high-throughput sequencing data analysis methods [128,129]. Many analysis methods are used for the analysis of scRNA-seq data, and the most commonly used methods are as follows:

1. Dimensionality reduction. Dimensionality reduction is actually achieved by combining several original features into some new feature information to obtain a set of compressed and refined feature information [130]. There are linear dimensionality reduction methods and nonlinear dimensionality reduction methods, and the dimensionality reduction methods based on linear decomposition models are principal component analysis, independent component analysis, weighted nonnegative matrix factorization, etc. [131]. Taking principal component analysis as an example, the method has been successfully applied in scRNA-seq data analysis [132–134] to capture the overall structure of cell heterogeneity, with the limitation that it cannot visualize the local structure necessary for cell clustering and cell type identification. Nonlinear dimensionality reduction methods are *t*-distributed stochastic neighbor embedding (*t*-SNE), uniform manifold approximation and projection [135], and *k*-nearest neighbor algorithm [136]. *t*-SNE is the most widely used data dimensionality reduction method in scRNA-seq data analysis [137].
2. Differential expression analysis. Differentially expressed gene analysis can detect mRNA abundance between different cell types and different cell subpopulations. The effect of different samples or different treatments on gene expression levels, either up- or down-regulated, can be obtained by intergroup comparison [138], which, in turn, allows functional analysis of differentially expressed genes. Differential expression algorithms for scRNA-seq have been developed successively, such as single-cell differential expression [139], MAST [140], Census [141], and BCseq [142].
3. Clustering analysis. Currently, the most popular clustering strategy in scRNA-seq data analysis is the community detection algorithm, and the representative algorithm is Louvain. There are also commonly used clustering strategies, including hierarchical clustering and partitioning clustering. For hierarchical clustering, representative algorithms include Agglomerative Nestling (AGNES) and Divisive Analysis (DIANA). The typical representative of segmentation clustering is the *k*-means algorithm [143]. In addition, density-based clustering methods (e.g., Density-Based Spatial Clustering of Applications with Noise), neural network-based clustering methods (e.g., self-organizing map), and model-based methods (e.g., expectation maximization algorithm) have also been applied to the field of scRNA-seq data analysis [144].
4. Trajectory inference analysis. In many biological systems, cells are not necessarily in a discrete state; instead, they exist in a continuous state. Some algorithms developed for scRNA-seq pseudotime analysis are Monocle [145], Waterfall [146], Tools for Single Cell Analysis (TSCAN) [147], Sincell [148], Selective Locally Linear Inference of Cellular Expression Relationships (SLICER) [149], Wishbone [150], Wanderlust [151], and single-cell clustering using bifurcation analysis (SCUBA) [152].
5. Prediction of cell communication. Network analysis is one of the most popular research strategies in the field of scRNA-seq, where cell–cell or gene–gene interactions and regulation exist all the time, and the construction of gene and cell-related interaction networks is essential to understand the molecular regulation mechanisms of complex biological traits at microscopic levels. Currently, there are gene co-expression networks, in which the weighted gene co-expression network is used as a classical network analysis method in scRNA-seq research [153]. Furthermore, cell–cell interaction networks are a type of network analysis, and many softwares are available to infer cell–cell interaction networks, including SingleCellSignalR, iTALK, NicheNet, celltalker, CellChat, CellPhoneDB, etc.

In recent years, scRNA-seq technology has emerged as a research method and has gradually become a hot research topic. In addition, scRNA-seq data analysis methods are widely used in the fields of stem cell, developmental biology, tumor, immunology, neurobiology, and skeletal system biology [154–157]. For example, Brunskill et al. [158] used single-cell sequencing to study the gene expression profile during kidney organ development and found that, at different developmental stages, cell populations with the same structure have different origins. Li et al. [159] sequenced 11 colorectal cancer cells and the corresponding normal mucosal cells using single-cell sequencing technology and identified 2 different fibroblastoma subtypes by clustering analysis using the reference component analysis algorithm, in which some up-regulated gene expressions associated with epithelial mesenchymal stem cell differentiation were also identified, providing a good method for characterizing tumor cell heterogeneity. Peng et al. [160] identified 10 types of cells in pancreatic cancer tissues by *t*-SNE analysis through single-cell transcriptome analysis of 24 preoperative human pancreatic cancer tissues without radiotherapy. The gene expression pattern of pancreatic ductal adenocarcinoma (PDAC) from precancerous to malignant state was investigated using trajectory analysis, and several classical oncogenic pathways, including ErbB and Notch signaling pathways, were found to be activated during the progression of PDAC. Song et al. [161] proposed PseudotimeDE, the first differential gene expression detection tool that takes into account the stochasticity of the pseudotime. This tool uses subsampling to help estimate the stochasticity of the pseudotime, a generalized additive model to fit the relationship between individual gene expression values and the pseudotime, and a permutation test to generate statistically rigorous *P* values. Compared to existing methods, PseudotimeDE has advantages in accounting for uncertainty in pseudotime inference.

## Discussion

Biological sequence classification is an important branch of biological informatics and is mainly related to the classification of DNA, RNA, and amino acid sequences. The goal of the classification task was to build a well-fitted and high-performance prediction model. In Tables S2–S4, we collected more than 100 classification tools for DNA, RNA, proteins, and peptides, as well as their predictive performance. We discuss the existing challenges and future opportunities in model construction for classification tasks with a focus on datasets, classification algorithms, and future trends in biological sequence classification.

### Dataset

The construction of a dataset is a key step in biological sequence classification research. The quality of the dataset is related to the performance of the constructed model. The prediction model built using high-quality datasets shows high predictive performance and generalization ability. Some datasets for biological sequence classification have the limitations of small data volumes and an imbalance between positive and negative samples (Tables S2–S4). In most biological sequence classification research, positive and negative samples are manually balanced. In biological informatics research, the process of obtaining datasets is expensive, time-consuming, and laborious. Therefore, positive and negative samples are usually not balanced in the acquired datasets. Data imbalance is a dataset with a positive–negative sample ratio of greater than 3 [35]. The number of positive samples may decrease in sequences analyzed with modern biological technology. For most datasets, a threshold of around 80% was used to remove homologous sequences, but this can lead to data leakage, which can lead to model overfitting and results that are better than they actually look. Therefore, it is necessary to reduce the homology threshold on the data to ensure that it is 25% to 30%.

In the procedure for dataset construction, less data and data imbalance are the major factors affecting data quality. In Table 2, we summarize methods using a small number of datasets and unbalanced datasets in RNA posttranscriptional modification research. For iRNA-m5C [162], the dataset used included four species (*H. sapiens*, *M. musculus*, *S. cerevisiae*, and *A. thaliana*), among which *H. sapiens* and *M. musculus* had smaller datasets (*H. sapiens*: P-120/N-120 and *M. musculus*: P-97/N-97). Using the jackknife test, the accuracy of *H. sapiens* and *M. musculus* was 100%. Because of the limitation of the number of sequences in the dataset, the accuracy of the model built for these 2 species was 100% and the model was overfitted. If a positive or negative sample is incorrectly predicted, then it will have a greater impact on the accuracy. Similarly, iRNA-PseKNC(2methyl) [163] and iRNA-2OM [53] were developed for Nm using the same dataset (*H. sapiens:* P-147/N-147). Under 5-fold CV, the Acc values obtained were 98.27% and 97.95%, respectively. In this type of biological sequence classification research, the dataset sequence is small, and there is no independent test set to verify the performance of the model; the accuracy obtained is high, but the model exhibits overfitting and poor migration. When the dataset increases the number of sequences, the prediction performance of the model decreases. The effect of ML is also poor because the sequence dataset and the sequence information obtained are limited. In the case of an imbalanced dataset, the iRNA-m5C_NB [99] predictor method was used, using only a *H. sapiens* dataset (Met935: training dataset: P-127/N-808; testing dataset: Test1157: P-157/N-1000). The effects of data imbalance were reduced by using SMOTEENN. The Acc values obtained by this method under CV and independent test sets were 82.20% and 74.85%, respectively. Similarly, Dou’s method [98] also adopts SMOTEENN for the imbalance of positive and negative samples in the D-modified dataset. The Acc values obtained using the jackknife test and independent test set were 97.24% and 93.75%, respectively. RFhy-m2G [24] classified m2G modifications and adopted SMOTE to deal with the problem of data imbalance. In addition to the data imbalance in the above method, the dataset was also small, and some positive samples have a limited number of sequences.

**Table 2.** Summary of 5 RNA posttranscriptional modification datasets.

Type	Method	Year	Dataset	Performance			Imbalance algorithms
				Evaluation	Acc (%)	AUC	
m5C	iRNA-m5C	2020	*H. sapiens*: TAD (P-120/N-120)	Jackknife test	90.80	0.963	/
			*M. musculus*: TAD (P-97/N-97)		100	1.000	
			*S. cerevisiae*: TAD (P-211/N-211)		100	1.000	
			*A. thaliana*: TAD (P-5289/N-5289); TSD (P-1000/N-1000)	Jackknife test	70.70	0.765	
				IDT	74.00	/	
	iRNA-m5C_NB	2020	*H. sapiens*: Met935: TAD (P-127/N-808); TSD (Test1157: P-157/N-1000)	Jackknife test	82.20	0.91	SMOTEENN
				IDT	74.85	0.83	
Nm	iRNA-PseKNC(2methyl)	2019	*H. sapiens*: TAD (P-147/N-147)	5-CV	98.27	/	/
	iRNA-2OM	2018			97.95	/	
m2G	RFhy-m2G	2021	*H. sapiens*: TAD (P-41/N-541); TSD (P-5/N-60)	5-CV	99.82	/	SMOTE
				IDT	94.17	/	
			*M. musculus*: TAD (P-27/N-427); TSD (P-3/N-47)	5-CV	100	/	
				IDT	90.43	/	
			*S. cerevisiae*: TAD (P-60/N-283); TSD (P-7/N-31)	5-CV	99.65	/	
				IDT	100	/	
D	Dou’s method	2021	Multispecies: TAD (P-140/N-298); TSD (P-36/N-76)	Jackknife test	97.24	0.99	SMOTEEEN
				IDT	93.75	0.87	

P, positive samples; N, negative samples; TAD, training dataset; TSD, testing dataset; 5-CV, 5-fold cross-validation; IDT, independent testing; SMOTE, synthetic minority oversampling technique; SMOTEENN, SMOTE and undersampling method edited nearest neighbors (ENN).

The limitations of datasets and data imbalances are prerequisites for constructing a high-quality dataset. (a) Because of the limitation of the dataset, the amount of data is small, and only by collecting more sequences from related databases or literature, it is best to be verified by experiments. (b) Data imbalance. The solution to the imbalance can be addressed in 3 steps [35,164]. First, the selection of the processing method should be based on the data itself, and positive and negative samples should be balanced at the sampling stage. At present, datasets are collated with various sampling methods, including undersampling, oversampling, and mixed sampling (combined undersampling and oversampling). Second, algorithm-based processing methods, which adjust ML algorithms or deep learning algorithms to correct data imbalances and improve model performance. The most commonly used method is pretraining. In addition, unsupervised pretraining and transfer learning can effectively deal with data imbalances. Third, a cost-sensitive learning method is commonly used to deal with the different costs of misclassified data in a single class. In this method, the objective loss function in deep learning is commonly used. Some researchers have directly used deep learning to study data imbalances. For unbalanced data, BACC was used to calculate the prediction accuracy.

### Classification algorithms

Supervised learning classification algorithms are the most commonly used and make up the core of biological sequence classification tasks. This type of algorithm is used to train a model on a sequence of known labels and find a well-performing model so that the new unknown sequence can be input for prediction. Commonly used classification algorithms for biological sequences are divided into traditional ML algorithms and deep learning algorithms:

1. The first is a commonly used classification algorithm (RF, SVM, NB, LR, DT, LGBM, and XGBoost; Fig. 1). Construction of the reliable model requires multiple iterations of experiments when using this model. There are more than a dozen traditional algorithms that are commonly used. To find a suitable classification algorithm, the researcher makes a preliminary selection based on experience or literature. This method will take a lot of time, and improper selection of the classification algorithm will affect the prediction results. For some biological sequences, when the prediction results of these traditional classification algorithms are not high, it is impossible to improve the prediction results by improving the algorithm itself. Integrating the weak classification algorithm with the strong classification algorithm only leads to a limited improvement in prediction results. Using traditional classification algorithms, most of the features used are handcrafted features, and the selection of suitable features among many feature coding methods will also be done manually, which influences the prediction performance of the model.
2. The second is the deep learning algorithm (basic neural networks, multilayer perceptrons, CNN, and RNN), which has been widely used in biological sequence classification tasks in recent years. Deep learning algorithms can directly input sequences and classify them by learning or combining manual features with automatic learning features. However, the process of deep learning algorithms is a black box, and it is impossible to explain in detail how it proceeds internally. For small datasets, the prediction effect of using deep learning algorithms is not as good as that of traditional classification algorithms. This may be due to insufficient sequence data for sequence information learning. Deep learning algorithms are suitable for larger datasets and deeper mining of sequence information when conducting biological sequence classification.

### Challenges and recommendations

With the continuous development of technologies in molecular biology, the availability of biological sequences has also increased. Computational biologists are required to develop more effective and efficient software for biomedical research. On the basis of this the summary work, we offer our thoughts on the challenges and recommendations for biological sequence classification field.

#### Dataset

First, we advise caution over the use of datasets in literature or collected by previous studies. Authors should check the datasets according to the assembly steps described in the original articles to ensure that they are correct. On the other hand, the construction procedure should be described adequately in the article if the datasets are being used for the first time. Overall, it is the responsibility of the researchers and authors to ensure that the data used for modeling and evaluation are appropriate. Furthermore, authors should analyze the quality and size of the training and testing datasets, and is there any source of bias, an often neglected but important point. On the basis of our survey, we noticed that some studies did not have sufficient test data or even enough training data. Without proper testing, it may not reflect the true performance of the model on unseen data, which undermines user confidence in the model.

#### Feature

ML algorithms are used to build a classification model based on sequence statistics to learn the input sequence features and output the prediction results. Various feature encoding methods have been developed [40] on the basis of methods such as sequence, structure, physical–chemical properties, and score matrix, but this is far from enough. Future priorities include not only developing feature descriptors that can contain more deeply hidden information but also exploring how to exploit known structural parts of biological systems, while using neural networks to learn unknown parts. In this way, many heavy models can be replaced by simpler models that are easier to interpret and more robust to unseen data. On the other hand, as mentioned in the “General scheme and principles of ML in biological sequence classification” section, development and selection of appropriate feature selection methods to obtain the optimization feature set are very important for the construction of a robust predictive model.

#### Standardization

With no standardization, ML algorithms for biological sequence classification have been developed with a very broad range of applications, dataset collection procedures, and performance evaluation metrics. Although the diversity of biological sequence data makes it difficult to develop common standards and guidelines, it will benefit the research community to establish even a few numbers of criteria for specific tasks, not only for authors but also for reviewers.

#### Collaboration

ML-based models can be thought as the interface between the data analysis and the wet experiment. Biomedical researchers are the prospective users of our developed computational tools. It is very important for computer scientists to collaborate with experimental biologists. Advances in predictive models require joint efforts from developers and users, and prompt feedback from even a small number of users can have a positive impact on model performance. Moreover, the collaboration will also help interpret the built models. Biologists are interested not only in just accurate modeling but also in discovering mechanisms and factors that determine model output. On the other hand, we recommend using reviewers with backgrounds in biology and ML, as they can judge technical and application aspects differently. In this way, models that produce robust predictions are ready to be deployed in real application scenarios.

#### Software

As described in the “General scheme and principles of ML in biological sequence classification” section, make the trained model long-term available in the form of a user-friendly web server in a suitable repository such as GitHub (https://github.com). This benefits both computational and biological experimental researchers as it allows them to build on the method without having to start from scratch. We noticed that many development servers were no longer accessible even after the article was just published. Additionally, some web servers do not explicitly describe usage guidelines and how their models are implemented. Therefore, we highly recommend the authors take the tool seriously after it is released and try to accommodate support requests.

## Conclusions

With the continuous development of biological technology, biological sequences have increased exponentially. Traditional biological experimentation techniques are time-consuming and laborious. Therefore, it is necessary to accurately classify these biological sequences on the basis of the computational tools developed by ML. In this review, we have summarized the data and general classification methods related to 3 types of biological sequences (DNA, RNA, and amino acids). An online webpage was also built to provide downloads of datasets, accuracy and AUC values of related methods, and a web server. In addition, we introduced the model construction process of biological sequence classification, including dataset collection, feature extraction and feature selection, ML algorithms, model performance evaluation, and web server implementation. The classification method based on DNA, RNA, and amino acid sequences was briefly introduced.

Although there are many general methods and related datasets for biological sequence classification, most of the methods are improved on the same dataset, and there is a lot of room for development. There are still challenges in biological sequence classification research tasks, such as small datasets and unbalanced data, and the interpretability and repeatability of ML algorithms. In order to fully understand more information about biological sequences, it is necessary to develop more classification methods and collect more biological sequence data. In the future, we will continue to update data and related methods to provide a public platform and benchmark data for biological sequence function and modification classification research to avoid data and method confusion. It is more helpful for researchers to have a simple understanding of the study of biological sequence classification. The discussion and analysis of the existing shortcomings of biological sequence research provides insights for future biological sequence classification research. For single-cell sequencing data analysis, we briefly introduce the commonly used methods and their applications in systems biology. In the future, we will give a more in-depth review of the hot research on single-cell sequencing data analysis.

## Supplementary Materials

Supplementary Materials Table S1. Biological sequence modification site database. Table S2. Summary of DNA sequence classification methods and dataset. Table S3. Summary of RNA sequence classification methods and dataset. Table S4. Summary of amino acid sequence classification methods and dataset.
