# Supplementary material for: Biological Sequence Classification: A Review on Data and General Methods
Source: Research (Wash D C). 2022 Dec 19;2022:0011. doi: 10.34133/research.0011 (PMC11404319; doi:10.34133/research.0011)
Supplement: Supplementary Materials — Table S1. Biological sequence modification site database. Table S2. Summary of DNA sequence classification methods and dataset. Table S3. Summary of RNA sequence classification methods and dataset. Table S4. Summary of amino acid sequence classification methods and dataset. [file research.0011.f1.pdf]

Biological sequence classification: a review on data and general methods

Chunyan Ao[1,2,3], Shihu Jiao[2], Yansu Wang[3], Liang Yu[1,*], Quan Zou[2,3,*]

1. School of Computer Science and Technology, Xidian University, Xi'an, China
2. Yangtze Delta Region Institute (Quzhou), University of Electronic Science and Technology of China, Quzhou, China
3. Institute of Fundamental and Frontier Sciences, University of Electronic Science and Technology of China, Chengdu, China

*Corresponding author: lyu@xidian.edu.cn, zouquan@nclab.net.

Contents

1 Supporting Tables

Table S1. Biological sequence modification site database S2-S3

Table S2. Summary of DNA sequence classification methods and dataset S3-S7

Table S3. Summary of RNA sequence classification methods and dataset S8-S17

Table S4. Summary of Amino acid sequence classification methods and dataset S18-S33

2 References S33-S41

Table S1. Biological sequence modification site database

Bio sequence	Dataset	Type	Website
DNA	MethDB	5mC	http://www.methdb.de
	MethSMRT	6mA, 4mC	http://sysbio.sysu.edu.cn/methsmrt/
	MDR		http://mdr.xieslab.org
	DNAmod	Multiple	https://dnamod.hoffmanlab.org
RNA	RMBase	Multiple	http://rna.sysu.edu.cn/rmbase/index.php
	RMDisease	Multiple	www.xjtlu.edu.cn/biologicalsciences/rmd
	RNAMDB	Multiple	http://rna-mdb.cas.albany.edu/RNAmods/[#]
	MODOMICS	Multiple	http://modomics.genesilico.pl
	RADAR	A-to-I RNA editing	http://RNAedit.com
	REDIportal		http://srv00.recas.ba.infn.it/atlas/
	MeT-DB	m6A	http://compgenomics.utsa.edu/methylation/
Protein	dbPTM	Multiple	http://dbptm.mbc.nctu.edu.tw/index.php
	SysPTM	Multiple	http://lifecenter.sgst.cn/SysPTM/
	PhosphoSitePlus	Multiple	http://www.phosphosite.org
	BioGRID	Multiple	https://thebiogrid.org/
	PhosphoNET	Phosphorylation	http://www.phosphonet.ca/
	EPSD		http://epsd.biocuckoo.cn/
	UniPep	Glycosylation	http://www.unipep.org/
	GlycoFish		http://betenbaugh.jhu.edu/GlycoFish
	GlycoFly		http://betenbaugh.jhu.edu/GlycoFly
	mUbiSiDa	Ubiquitylation	http://reprod.njmu.edu.cn/mUbiSiDa
	SwissPlam	S-plamitoylation	https://swisspalm.org/
	dbSNO	S-nitrosylation	http://dbSNO.mbc.nctu.edu.tw

Peptide	StarPep	general	http://isyslab.info/StraPep/
	FeptideDB	general	http://www4g.biotec.or.th/FeptideDB/index.php
	AntiTbPdb	Antitubercular	https://webs.iiitd.edu.in/raghava/antitbpdb/
	CPPsite/CPPsite 2.0	Cell penetrating	https://webs.iiitd.edu.in/raghava/cppsite/
	CancerPPD	Anticancer	http://crdd.osdd.net/raghava/cancerppd/

Note: # Website is not available at the time of writing.

Table S2. Summary of DNA sequence classification methods and dataset.

Type	Method	Year	Classifier	Dataset			Performance		URL
				TAD: P/N	TSD: P/N	Imbalance Algorithm	Evaluation	Acc	
DNA Enhancers	iEnhancer-RF[1]	2021	RF	(S+W)-742+742/1484	(S+W)-100+100/200	/	5-CV	LayerI:76.18%; LayerII:62.53%	http://nsclbio.jbnu.ac.kr/tools/iEnhancer-RF/
							IDT	**LayerI:79.75%; LayerII:85.00%**	
	iEnhancer-XG[2]	2021	XGBoost				10-CV	**LayerI:81.10%; LayerII:66.74%**	https://github.com/jimmyrate/ienhancer-xg
							IDT	LayerI:75.75%; LayerII:63.50%	
	iEnhancer-EL[3]	2018	Ensemble Learning				JKT	LayerI:78.03%; LayerII:65.03%	http://bioinformatics.hitsz.edu.cn/iEnhancer-EL/
							IDT	LayerI:74.75%; LayerII:61.00%	
	EnhancerPred[4]	2016	SVM		/		JKT	LayerI:77.39%; LayerII:68.19%	http://server.malab.cn/EnhancerPRED/
	iEnhancer-2L[5]	2015	SVM				JKT	LayerI:76.89%; LayerII:61.93%	http://bioinformatics.hitsz.edu.cn/iEnhancer-2L/
Promoters	iPSW(2L)-PseKNC[6]	2019	SVM	3382/3382	/	/	5-CV	84.06%	http://www.jci-bioinfo.cn/iPSW(2L)-PseKNC
	iPro2L-PSTKNC[7]	2021	SVM	σ^{24}: 484; σ^{28}: 134; σ^{32}: 291; σ^{38}: 163; σ^{54}: 94; σ^{70}: 1694/2860		SMOTE	5-CV	**LayerI: 90.05%, LayerII: σ24: 97.75%; σ28: 99.84%; σ32: 98.66%;**	https://github.com/lyuyinuo/iPro2L-PSTKNC

								**σ38: 99.06%; σ54: 99.94%; σ70: 94.19%**	
	iPromoter-2L[8]	2018	RF			IHTS	5-CV	LayerI: 81.68%, LayerII: σ24: 93.50%; σ28: 96.85%; σ32: 94.41%; σ38: 94.69%; σ54: 94.04%; σ70: 80.66%	http://bioinformatics.hitsz.edu.cn/iPromoter-2L/
Nucleosome	NucPosPred[9]	2018	SVM GBDT	*C. elegans:* 2567/2608; *D. melanogaster:* 2900/2850		/	JKT	C: 92.29%; D: 88.26%	http://121.42.167.206/NucPosPred/index.jsp
Functional of DNA sequence	DeepATT[10]	2021	DNN	440000(P/N=1/1) Validation: 8000	Total:455024	/	/	AVAUROC: 0.94519	https://github.com/jiawei6636/Bioinfor-DeepATT
N4-methycytosine (4mC)	4mC-w2vec[11]	2021	CNN	*F. vesca:* 3457/3457 *R. chinensis*: 1938/1938	*F. vesca*: 864/864,4320,12960 *R. chinensis:* 483/2415,7245	/	5-CV	***F.vesca*: 86.97%; *R.chinensis:* 85.41%**	http://nsclbio.jbnu.ac.kr/tools/4mC-w2vec/
							IDT	*F.vesca*: (P/N=1:1 86.32%; P/N=1:5 86.32%; P/N=1:15 84.12%); *R.chinensis:* (P/N=1:1 84.90%; P/N=1:5 84.00%; P/N=1:15 84.77%);	
	EC4mC-SVM[12]	2020	SVM	388/388	134/134	/	10-CV	85.4%	/
							IDT	83.2%	
	Deep4mcPred[13]	2020	DNN	*A. thaliana*: 20000/20000; *C. elegans:* 20000/ 20000; *D. melanogaster:* 20000/ 20000;		/	10-CV	*A. thaliana*: 84.4%; *C. elegans*: 89.3%; *D. melanogaster*: 87.1%;	http://server.malab.cn/Deep4mcPred
	4mcPred-IFL[14]	2019	SVM	*C. elegans:* 1554 /1554; *D. melanogaster*: 1769 /1769; *A. thaliana:* 1978 /1978; *E. coli:* 388/388; *G. subterraneus*: 905/905;		/	Cross-validation	***C. elegans:* 88.00%; *D. melanogaster*: 87.40%; *A. thaliana:* 82.50%; *E. coli*: 89.40%; *G. subterraneus*: 88.60%; *G. pickeringii*: 90.70%;**	http://server.malab.cn/4mcPred-IFL/
	4mcPred-	2019	SVM			/	10-CV	*C.elegans*: 81.50%; *D.melanogaster*:	http://server.malab.cn/4mcPred-SVM

	SVM[15]			*G. pickeringii:* 569/569;				83.00%; *A.thaliana*: 78.70%; *E.coli*: 83.30%; *G.subterruneus*: 83.70%; *G.pickeringii:* 86.00%	
	4mCPred[16]	2019	SVM			/	JKT	*C.elegans*: 87.71%; *D.melanogaster*: 87.79%; *A.thaliana*: 83.37%; *E.coli*: 94.97%; *G.subterruneus*: 91.04%; *G.pickeringii*: 90.89%	http://server.malab.cn/4mCPred/index.jsp
							IDT	*C.elegans*: 82.21%; *D.melanogaster*: 82.63%; *A.thaliana*: 76.52%; *E.coli*: 82.69%; *G.subterruneus*: 83.33%; *G.pickeringii:* 77.63%	
	Meta-4mCpred[17]	2019	SVM	*C. elegans:* 1554 /1554; *D. melanogaster*: 1769 /1769; *A. thaliana:* 1978 /1978; *E. coli:* 388/388; *G. subterraneus*: 905/905; *G. pickeringii:* 569/569;	*C. elegans*: 750/750; *D. melanogaster:* 1000/ 1000; *A. thaliana:* 1250/ 1250; *E. coli*: 134/134; *G. subterraneus*: 350/350; *G. pickeringii*: 200/200;	/	10-CV	*C.elegans:*82.60%; *D.melanogaster*: 84.20%; *A.thaliana*: 79.20%; *E.coli*: 84.80%; *G.subterruneus*: 85.50%; *G.pickeringii*: 89.10%	http://thegleelab.org/Meta-4mCpred
							IDT	*C.elegans:* 87.00%; *D.melanogaster:* 90.60%; *A.thaliana:* 85.50%; *E.coli:* 82.50%; *G.subterruneus:* 85.00%; *G.pickeringii:* 85.00%	
	4mCpred-	2019	RF\GBDT\	800/800	180/180	/	10-CV	79.50%	http:/thegleelab.org/4mCpred-EL

	EL[18]		SVM\ERT				IDT	79.80%	
N6-methyladenine (6mA)	MGF6mARice[19]	2022	MLP	Rice-Chen: 880/880<br>Rice-Lv: 154000/154000<br>Imbalanced:<br>Rice-Chen (1:5):176/880<br>Rice-Chen (1:10):88/880<br>Rice-Chen (1:20):44/880<br>Rice-Lv(1:5):30800/154000<br>Rice-Lv(1:10):15400/154000<br>Rice-Lv(1:20):77--/154000	NIP: 10000/10000<br>*A.* thaliana: 15937/15937<br>*D.melanogaster:* 11191/11191<br>*R. chinensis:* 11815/11815	/	10-CV	Rice-Chen: 97.00%<br>Rive-Lv: 96.00%	https://github.com/zhlSunLab/MGF6mARice
							IDT	NIP_10000: 88.00%<br>*A.* thaliana: 77.00%<br>*D.melanogaster:* 82.00%<br>*R. chinensis:* 86.00%	
	Deep6mA[20]	2021	CNN<br>LSTM	*Rice:* 154000/154000<br>*A. thaliana:* 98483/98483<br>*F. vesca:* 1417/1417<br>*R. chinensis:* 5733/5733		/	5-CV	*Rice:* ACC 94.01%<br>*A. thaliana:* AUC 0.954<br>*F. vesca:* AUC 0.982<br>*R. chinensis:* 0.961	https://github.com/Marscolono/Deep6mA

	iRicem6A-CNN[21]	2021	CNN	154000/154000	880/880	/	5-CV	**93.82%**	http://iRicem6ACNN.aibiochem.net
							IDT	**96.19%**	
	6mA-RicePred[22]	2020	SVM	880/880	154000/154000	/	10-CV	87.27%	https://github.com/huangqianfei0916/6marice/tree/master/dataset
							IDT	85.65%	
	iIM-CNN[23]	2019	CNN	*cross-species:* 2768/2716; *Rice:* 880/880; *M. musculus:* 1934/1934;		/	5-CV	*cross-species:* 82.40%; *Rice:* 87.50%; *M.musculus:* 96.90%	https://home.jbnu.ac.kr/NSCL/iIMCNN.htm
	csDMA[24]	2019	ExtraTrees	*Cross-species: 2214/2214; Rice:* 880/880; *M.musculus:* 1934/1934	*Cross-species: 554/554*	/	5-CV	*cross-species:* 79.90%; *Rice:* 86.10%; *M.musculus:* 96.60%;	https://github.com/liuze-nwafu/csDMA
							IDT	*cross-species:* 81.30%	
5-methylcytosine (5mC)	iPromoter-5mC[25]	2020	DNN	55800/13950	658861/164715	DSM	5-CV	90.16%	http://www.jci-bioinfo.cn/iPromoter-5mC
							IDT	90.22%	
	iDNA - Methyl[26]	2015	SVM	787/1639		NCR/SMOTE	JKT	77.49%	http://www.jci-bioinfo.cn/iDNA-Methyl

Note: # URL is not available. P: positive samples; N: negative samples; TAD: Training dataset; TSD: Testing dataset; 5-CV: 5-fold cross validation; 10-CV: 10-fold cross validation; IDT: Independent testing; JKT: jackknife test. SMOTE: Synthetic Minority Oversampling Technique; IHTS: Inserting Hypothetical Training Samples; GBDT: gradient-boosting decision tree; SVM: support vector machine; RF: random forest; DNN: deep neural networks; CNN: convolutional neural network; ERT: extremely randomized tree; XGBoost: extreme gradient boosting;

Table S3. Summary of RNA sequence classification methods and dataset.

Type	Method	Year	Classifier	Dataset			Performance		URL
				TAD: P/N	TSD: P/N	Imbalance Algorithm	Evaluation	Acc	
eukaryotic mRNA	SubLocEP[27]	2021	LightGBM	Cytoplasm: 5310; Endoplasmic reticulum: 1185; Extracellular region: 710; Mitochondria: 350; Nucleus: 4855;	D1: Cytoplasm: 1066; Endoplasmic reticulum: 241; Extracellular region: 145; Mitochondria: 71; Nucleus: 976; D2: Cytosol: 91; Nucleus: 148; D3: Endoplasmic reticulum: 131; Nucleus: 131;	/	5-CV	65.90%	http://lab.malab.cn/~lijing/SubLocEP.html
							IDT	D1: 60.10%<br>D2: 50.60%<br>D3: 37.0%	
lncRNA	PlncRNA-HDeep[28]	2021	LSTM<br>CNN	18000/18000;<br>(training set (80%) and test data (20%))		/	5-CV	96.5%	https://github.com/kangzhai/PlncRNA-HDeep
	PredLnc-GFStack[29]	2019	RF	Human Main: 35760/20299<br>Mouse Main: 23987/11746	Human Independent: 1500/1500; Mouse Independent: 1500/1500; CPPred (See online)	/	10-CV	**Human-Main: 0.895;**<br>**Mouse-Main: 0.914;**	https://github.com/BioMedicalBigDataMiningLab/PredLnc-GFStack/
							IDT	**Human-Main:**<br>Human-Testing: 0.968; Mouse-Testing: 0.941; Zebrafish-Testing: 0.901; Fruit-fly-Testing: 0.940; S.cerevisiae-Testing: 0.960; Integrate-Testing: 0.907;	

								**Mouse-Main:**<br>Human-Testing: 0.887; Mouse-Testing: 0.944; Zebrafish-Testing: 0.843; Fruit-fly-Testing: 0.917; S.cerevisiae-Testing: 0.942; Integrate-Testing: 0.871;	
Non-Coding RNAs	ncRFP[30]	2021	Deep learning	6320 ncRNAs sequence<br>[**PS**: all ncRNAs sequences can form 10-fold cross-validation train sets and test sets]		/	10-CV	0.7972	https://github.com/linyuwangPHD/ncRFP
	ncRDense[31]	2021	Deep learning	6320	2600	/	10-CV	**0.9519**	http://nsclbio.jbnu.ac.kr/tools/ncRDense/
							IDT	**09510**	
	ncRDeep[32]	2020	CNN			/	10-CV	88.40%	https://home.jbnu.ac.kr/NSCL/ncRDeep.htm
pre-miRNA	ASRmiRNA[33]	2022	SVM	miRNA dataset: 376/376;<br>Pre-miRNA dataset: 251/251;<br>Pre-miRNA+miRNA dataset: 251 stress-responsive Pre-miRNAs/251 non-stress-responsive Pre-miRNAs; 251 stress-responsive miRNAs/251 non-	70 stress-responsive miRNAs/100 non-stress-responsive miRNA;<br>70 stress-responsive Pre-miRNAs/100 non-stress-responsive Pre-miRNA	/	5-CV	miRNA: 65.33%;<br>Pre-miRNA: 66.40%;<br>miRNA + Pre-miRNA: 71.40%;	http://cabgrid.res.in:8080/asrmirna
							IDT	miRNA: 62.33%;<br>Pre-miRNA: 64.85%;<br>miRNA + Pre-miRNA: 69.21%;	

				stress-responsive miRNAs;					
	DNNPreMiR[34]	2020	DNN	Training dataset: 2408 sequences; Validation dataset: 602 sequences;	752 sequences	/	10-CV	CNN: 87.24±1.80%; RNN: 88.44±1.80%	https://github.com/zhengxueming/dnnPreMiR
circRNA	CirRNAPL[35]	2020	ELM	circRNA vs PCG: 10000/8000; circRNA vs lncRNA: 10000/10000; Stem cell vs not: 1800/1800;	circRNA vs PCG: 4084/1533; circRNA vs lncRNA: 4084/9722; Stem cell vs not: 282/282;	/	10-CV	circRNA vs PCG: 81.50%; circRNA vs lncRNA: 80.20%; Stem cell vs not: 78.20%;	http://server.malab.cn/CirRNAPL/
							IDT	circRNA vs PCG: 82.70%; circRNA vs lncRNA: 85.40%; Stem cell vs not: 81.20%;	
tRNA	tRNA-Predict[36]	2015	ensemble classifier	623/1183		/	10-CV	95.10%	http://datamining.xmu.edu.cn/~gjs/tRNA-Predict[#]
piRNA	2S-piRCNN[37]	2020	CNN	First Layer: $S^{+}_{inst+}/S^{+}_{non-inst}$= 709/709 Second Layer: S^{+}/S^{-}= 1418/1418		/	5-CV	First Layer: **93.59%** Second Layer: **90.13%**	http://nsclbio.jbnu.ac.kr/tools/2S-piRCNN/
	2L-piRNA[38]	2017	SVM			/	5-CV	First Layer: 86.1% Second Layer: 77.6%	http://bioinformatics.hitsz.edu.cn/2L-piRNA/
sgRNA	SgRNA-RF[39]	2021	RF	G17 sequences: 830/900; Gr sequences: 550/320;	G17 sequences: 229/3351; Gr sequences: 181/118;	CS-Smote	10-CV	G17: 84.7%; Gr: 69.4%; G5: hct116: 96.9%; hek293t: 94.0%; hela: 97.74%; h160: 94.10%;	https://server.malab.cn/sgRNA-RF/
							IDT	G17: 86.3%;	

				Gnr sequences: 536/180;<br>Gm sequences: 664/180;<br>G5 sequences:<br>hek293t:1615/428;<br>hct116: 3090/428;<br>hela: 5923/428;<br>h160:1973/428;	Gnr sequences: 135/57;<br>Gm sequences: 166/51;<br>G5 sequences:<br>hek293t:404/108;<br>hct116:783/108;<br>hela:782/108;<br>h160:494/108;			Gr: 91.6%;<br>Gnr: 89.4%;<br>Gm: 93.8%;<br>G5: hct116: 96.50%; hek293t: 78.70%;<br>hela: 97.90%;<br>h160: 97.30%;	
multi-type modification	MultiRM[40]	2021	DNN	m6A: 65178; Pseudouridine: 3137; m1A: 16380; m6Am: 2447; Am: 1591; Cm: 1878; Gm: 1471; Um: 2253; m5C: 12936; m7G: 1036; m5U: 1696; I: 52618;		OHEM, UW	/	Am: 78.00%; Cm: 82.00%; Gm: 89.00%; Um: 82.00%; m1A: 72.00%; m5C: 85.00%; m5U: 92.00%; m6A: 80.00%; m6Am: 83.00%; m7G: 65.00%; Pseudouridine: 84.00%; I: 70.00%;	www.xjtlu.edu.cn/biologicalsciences/multirm
	iMRM[41]	2020	XGboost	***H. sapiens:*** m1A: 6366/6366; m5C: 120/120; m6A: 1130/1130; Pseudouridine: 495/195; A-to-I: 3000/3000;<br>***S. cerevisiae:*** m1A: 483/483; m5C: 211/211; m6A: 1307/1307; Pseudouridine: 313/314;<br>***M. musculus:*** m1A: 1064/1064; m5C: 97/97; m6A: 725/725; Pseudouridine: 472/472;		/	10-CV	***H. sapiens:*** (m1A: 99.47%; m5C: 93.33%; m6A: 91.28%; Pseudouridine: 66.47%; A-to-I: 91.73%);<br>***S. cerevisiae:*** (m1A: 98.87%; m5C: 100%; m6A: 78.41%; Pseudouridine: 71.91%);<br>***M. musculus:*** (m1A: 99.29%; m5C: 99.00%; m6A: 89.59%; Pseudouridine: 74.48%);	http://www.bioml.cn/XG_iRNA/home

						/	JKT	***H. sapiens:*** (m1A: 99.41%; m5C: 92.08%; m6A: 91.02%; Pseudouridine: 64.55%; A-to-I: 91.57%);<br>***S. cerevisiae:*** (m1A: 98.86%; m5C: 99.52%; m6A: 77.77%; Pseudouridine: 71.08%);<br>***M. musculus:*** (m1A: 99.20%; m5C: 98.45%; m6A:88.97%; Pseudouridine: 73.09%);	
Pseudouridine	RF-PseU[42]	2020	RF	*H. sapiens:* 495/495;<br>*S. cerevisiae:* 314/314;<br>*M. musculus:*472/472;	*H.sapiens:*100/100;<br>*S.cerevisiae:*100/100;	/	10-CV	***H. sapiens*: 64.30%;**<br>***S. cerevisiae*: 74.80%;**<br>***M. musculus*:74.80%;**	http://rfpsu.aibiochem.net/
							IDT	***H. sapiens*: 75.00%;**<br>***S. cerevisiae*: 77.00%;**	
	iPseU-CNN[43]	2019	CNN			/	5-CV	*H. sapiens*: 66.68%;<br>*S. cerevisiae*: 68.15%;<br>*M. musculus*:71.81%;	/
							IDT	*H. sapiens*: 69.00%<br>*S. cerevisiae*: 73.50%	
	Dou’s method[44]	2020	RF\SVM	*H. sapiens:*495/495;<br>*S. cerevisiae:*319/319;<br>*M. musculus:*495/495;	*H.sapiens:*100/100;<br>*S.cerevisiae:*319/319;	/	5-CV	*H. sapiens*: 62.73%;<br>*S. cerevisiae*: 70.54%;<br>*M. musculus*: 71.72%;	/
							IDT	*H. sapiens*: 60.20%<br>*S. cerevisiae*: 77.0%	

5-methylcytosine (m5C)	Dou's method[45]	2020	SVM	6289/6289	1000/1000	/	10-CV	73.60%	/
							IDT	80.15%	
	iRNA-m5C[46]	2020	RF	*H.sapiens:* 120/120 ; *M. musculus:* 97/97; *S. cerevisiae:* 211/ 211; *A. thaliana:* 5289/5289;	*A. thaliana:* 1000/1000	/	JKT	*H. sapiens:* 90.80%;<br>*M. musculus:* 100%;<br>*S. cerevisiae:* 100%;	http://lin-group.cn/server/iRNA-m5C/service.html
							10-CV (*A. thaliana*)	*A.thaliana:* 70.70%	
							IDT	*A.thaliana:* 74.00%	
	iRNA-m5C_NB[47]	2020	NB	127/808	157/1000	SMOTEENN	JKT	82.20%	/
							IDT	74.85%	
5-hydroxymethylcytosine (5hmC)	iRhm5CNN[48]	2021	CNN	662/662		/	5-CV	**81.00%**	http://nsclbio.jbnu.ac.kr/tools/iRhm5CNN/
	iRNA5hmC[49]	2020	SVM			/	5-CV	65.48%	http://server.malab.cn/iRNA5hmC
N6-methyladenosine (m6A)	DNN-m6A[50]	2021	DNN	*Human*<br>**Brain:** 4605/4605;<br>**Kindey:** 4574/4574;<br>**Liver:** 2634/2634;<br>*Mouse*<br>**Brain:** 8025/8025;<br>**Heart:** 2201/2201;<br>**Kidney:** 3953/3953;<br>**Liver:** 4133/4133;<br>**Testis:** 4704/4704;	*Human*<br>**Brain:** 4604/4604;<br>**Kindey:** 4573/4573;<br>**Liver:** 2634/2634;<br>*Mouse*<br>**Brain:** 8025/8025;<br>**Heart:** 2200/2200;<br>**Kidney:** 3952/3952;<br>**Liver:** 4133/4133;<br>**Testis:** 4706/4706;	/	5-CV	***Human:*** **(Brian: 73.78%; Kidney: 80.48%; Liver: 81.30%);**<br>***Mouse:*** **(Brian: 79.36%; Heart: 76.17%; Kidney: 81.96%; Liver: 73.58%; Testis: 76.62%);**<br>***Rat*:** **(Brain: 78.27%; Kidney: 83.38%; Liver: 82.63%);**	https://github.com/GD818/DNN-m6A
							IDT	***Human:*** **(**Brian: **73.27%;** Kidney: **79.89%;** Liver: **80.96%);**<br>***Mouse:*** (Brian: **78.59%;** Heart: **51.10%;** Kidney: **80.87%;** Liver: **72.95%;** Testis: **77.12%);**	

				Rat<br>**Brain:** 2352/2352;<br>**Kidney:**<br>3433/3433;<br>**Liver:** 1762/1762;	*Rat*<br>**Brain:** 2351/2351;<br>**Kidney:**<br>3432/3432;<br>**Liver:** 1762/1762;			***Rat*: (**Brain: **77.99%;** Kidney: **83.04%;** Liver: **81.64%);**	
	iRNA-m6A[51]	2020	SVM			/	5-CV	*Human:* (Brian: 71.26%; Kidney: 78.99%; Liver: 80.13%);<br>*Mouse:* (Brian: 78.75%; Heart: 72.79%; Kidney: 79.98%; Liver: 70.59%; Testis: 74.40%);<br>*Rat:* (Brain: 75.96%; Kidney: 81.78%; Liver: 80.90%);	http://lin-group.cn/server/iRNA-m6A/
							IDT	*Human:* (Brian: 71.1%; Kidney: 77.76%; Liver: 79.01%);<br>*Mouse:* (Brian: 78.26%; Heart: 71.3%; Kidney: 79.31%; Liver: 68.79%; Testis: 73.54%);<br>*Rat*: (Brain: 75.14%; Kidney: 81.42%; Liver: 79.85%);	
	HSM6AP[52]	2021	XGBoost	14025/14025	D1: 23478/23478;<br>D2: 40742/40742;<br>D3: 15696/15696;	/	5-CV	**Full transcript:**<br>**A549: 97.30%; CD8T: 96.80%; HEK293-abacm: 97.80%; HEK293_sysy: 98.50%; Hela: 98.40%; MOLM13: 96.80%;**<br>**Mature:**<br>**A549: 90.80%; CD8T: 89.00%; HEK293-abacm: 97.00%; HEK293_sysy: 89.90%; Hela: 90.10%; MOLM13: 96.70%;**	http://lab.malab.cn/~lijing/HSM6AP.html

							IDT	**D1:**<br>**Full transcript:** AUC: **0.976;**<br>**Mature mRNA:** AUC: **0.899;**<br>**D2:**<br>**Full transcript:** AUC: **0.981;**<br>**Mature mRNA:** AUC: **0.914;**<br>**D3:**<br>**Full transcript:** AUC: **0.96776;**<br>**Mature mRNA:** AUC: **0.890;**	
	WHISTLE[53]	2019	SVM			/	5-CV	Full transcript: AUC (A549: 0.977; CD8T: 0.976; Hela: 0.976; HEK293(sys): 0.976; HEK293(abacm): 0.975; MOLM13: 0.979;)<br>Mature mRNA: AUC (A549: 0.938; CD8T: 0.940; Hela: 0.936; HEK293(sysy): 0.941; HEK293(abacm): 0.942; MOLM13: 0.943);	https://whistle-epitranscriptome.com/
							IDT	Full Transcript: **AUC** (A549: 0.973; CD8T: 0.947; Hela: 0.962; HEK293(sysy): 0.954; HEK293(abacm): 0.976; MOLM13: 0.947);<br>Mature mRAN: **AUC** (A549: 0.923; CD8T: 0.922; Hela: 0.911;	

								HEK293(sysy): 0.904; HEK293(abacm): 0.857; MOLM13: 0.856);	
	iMethyl-Deep[54]	2020	CNN	D1: M6A2614: 1307/1307<br>D2: M6A6540: 3270/3270		/	10-CV	**D1: 89.19%; D2: 87.44%**	https://github.com/abdul-bioinfo/iMethyl-deep
	DeepM6APred[55]	2018	SVM			/	10-CV	D1: 80.50%	http://server.malab.cn/DeepM6APred/[#]
	RAM-ESVM[56]	2016	SVM			/	JKT	D1: 78.35%	http://server.malab.cn/RAM-ESVM/[#]
	HLMethy[57]	2019	SVM	11799/11799	2976/2976	/	5-CV	72.60%	https://github.com/NWAFU-LiuLab/HLMethy
							IDT	72.70%	
	Gene2vec[58]	2018	CNN	88579(P/N=1/1)	88227(P/N=1/1)	/	10-CV	*H. sapiens:* **AUC** 0.8414;<br>*M. musculus:* **AUC** 0.8145;	http://server.malab.cn/Gene2vec/
							IDT	**AUC** 0.8414	
	BERMP[59]	2018	Deep learning	*S.cerevisiae:*1307/1307;<br>*A. thaliana:*2100/2100;<br>*Mammalian:*40000/400000;	*S.cerevisiae:*207/207;<br>*A. thaliana:*418/418;<br>*Mammalian:*10000/100000;	/	10-CV	*S. cerevisiae:* 71.26%;	http://www.bioinfogo.org/bermp/
							5-CV	*A. thaliana:* 85.95%;<br>*Mammalia:*<br>Full transcript: 87.80%; Mature mRNA: 86.14%;	
							IDT	*S. cerevisiae:* 68.84%;<br>*A. thaliana:* 87.20%;<br>*Mammalia:*<br>Full transcript: 88.34%;<br>Mature mRNA: 86.32%;	
2’-O-methylation	NmRF[60]	2022	RF	H.sapiens: 215/215;<br>S.cerevisiae:	H.sapiens: 46/114	/	10-CV	*H. sapiens*: 89.069%;<br>*S.cerevisiae*: 93.885%;	http://39.100.246.211:7001/

				89/189; M.musculus: 10/35;			IDT	*H. sapiens:* 86.88%	
	iRNA-PseKNC(2methyl)[61]	2019	CNN	147/147		/	5-CV	**98.27%**	/
	iRNA-2OM[62]	2018	SVM			/	5-CV	97.95%	http://lin-group.cn/server/iRNA-2OM/
N2-methylguanosine (m2G)	RFhy-m2G[63]	2021	RF	*H. sapiens:* 41/541; *M. musculus:* 27/427; *S.cerevisiae:*60/283 ;	*H. sapiens:* 5/60; *M. musculus:* 3/47; *S. cerevisiae:*7/31;	SMOTE	5-CV	*H. sapiens:* 99.82%; *M. musculus:* 100%; *S. cerevisiae:* 99.65%;	/
							IDT	*H. sapiens:* 94.17%; *M. musculus:* 90.43%; *S. cerevisiae:* 100%;	
Dihydrouridine (D)	iRNAD_XGBoost [64]	2021	XGBoost	140/298	36/76	SMOTEEEN	JKT	97.24%	/
							IDT	93.75%	
N7-methylguanosine (m7G)	m7G-DPP[65]	2021	SVM	741/741		/	10-CV	**95.42%**	https://figshare.com/articles/online_resource/m7G-DPP/15000348
							JKT	**95.55%**	
	m7G-IFL[66]	2020	XGBoost			/	10-VC	92.50%	http://server.malab.cn/m7G-IFL/
	iRNAm7G[67]	2019	SVM			/	JKT	89.88%	http://lin-group.cn/server/iRNAm7G/
	m7GPredictor[68]	2020	SVM	595/595	150/150	/	10-CV	86.00%	https://github.com/NWAFU-LiuLab/m7Gpredictor
							IDT	86.00%	

Note: # URL is not available. P: positive samples; N: negative samples; TAD: Training dataset; TSD: Testing dataset; 5-CV: 5-fold cross validation; 10-CV: 10-fold cross validation; IDT: Independent testing; JKT: jackknife test. SMOTE: Synthetic Minority Oversampling Technique; SMOTEENN: Synthetic Minority Over-sampling Technique (SMOTE) and under sampling method Edited Nearest Neighbours (ENN); OHEM: online hard examples mining; UW: uncertain weights; SVM: support vector machine; RF: random forest; DNN: deep neural networks; CNN: convolutional neural network; ELM: extreme learning machine; NB: Naive Bayes; XGBoost: extreme gradient boosting; LightGBM: light gradient boosting; LSTM: long short-term memory;

Table S4. Summary of Amino acid sequence classification methods and dataset.

Type	Method	Year	Classifier	Dataset			Performance		URL
				TAD: P/N	TSD: P/N	Imbalance Algorithm	Evaluation	Acc	
**(I) Protein**									
DNA-Binding Proteins	TargetDBP plus[69]	2021	SVM	4500/4500	381/381	/	JKT	86.84%	https://csbio.njust.edu.cn/bioinf/targetdbpplus/[#]
							IDT	85.83%	
	PSSMEI[70]	2020	SVM	525/525	93/93	/	JKT	86.05%	http://eie.usts.edu.cn/prj/PSSMEI/index.html
							IDT	75.30%	
Gram-negative bacteria	BastionHub[71]	2021	HMM	T1SS: 195; T2SS: 83; T3SS: 1194; T4SS: 713; T6SS: 181;		/	/	/	https://bastionhub.erc.monash.edu/
Bacterial Type III Secreted Effectors	iT3SE-PX[72]	2021	SVM	379/1112	108/108	/	5-CV	0.967±0.002	https://github.com/taigangliu/iT3SE-PX
							IDT	0.963±0.034	
	T3SEpp[73]	2020	Deep learning	309/310	42/34	/	10-CV	0.941±0.011	http://www.szu-bioinf.org/T3SEpp/index.html
							IDT	0.83	
Soluble protein	NetSolP[74]	2022	Deep learning	5718/5718	1550/1550	/	5-CV	0.70±0.02	https://services.healthtech.dtu.dk/service.php?NetSolP
							IDT	**0.728**	
	SoluProt[75]	2021	GBM			/	5-CV	**76.0%**	https://loschmidt.chemi.muni.cz/soluprot/
							IDT	58.5%	
Non-classical secreted proteins (NCSPs)	ASPIRER[76]	2021	XGBoost\CNN	141/446	34/34	SMOTE	5-CV	0.896±0.019	https://github.com/yanwu20/ASPIRER
							IDT	0.8088	
	NonClasGP-Pred[77]	2020	Deep learning			/	10-CV	**93.23%**	http://lab.malab.cn/~wangchao/softwares/NonClasGP/
							IDT	**86.76%**	
	PeNGaRoo[78]	2020	LightGBM			/	5-CV	0.900±0.016	https://pengaroo.erc.monash.edu/
							IDT	0.779	

Type IV secreted effector proteins	T4SEfinder[79]	202	MLP	518/1584	20/150	/	5-CV	90.4±1.4%	https://tool2-mml.sjtu.edu.cn/T4SEfinder_TAPE/
							IDT	96.5%	
	iT4SE-EP[80]	2021	SVM	T4SE:390/1112; Train915:305/610;	T4SE: 30/150; Train915:75/775;	/	JKT	Train915: 0.924; Train1502 0.950;	https://github.com/taigangliu/iT4SE-EP
							IDT	Test850: 0.956; Test180: 0.966;	
	Bastion4[81]	2019	ensemble learning	390/1112	30/150	/	5-CV	0.957±0.025	https://bastion4.erc.monash.edu/
							IDT	0.953±0.014	
Secretory Proteins	CRCF[82]	2021	RF	252/252		/	10-CV	97.80%	http://www.labio.info/optiRaac/
	SecProMTB[83]	2019	SVM	35/266		SMOTE	10-CV	91.60%	http://server.malab.cn/SecProMTB/index.jsp
							IDT	86.00%	
Cancerlectin	Tang's method[84]	2021	SVM	Cancerlectin: 176/227 Lectins: 462/435		/	5-CV	Cancerlectin: 98.54% Lectin: 95.38%	/
	Cancerlectins[85]	2020	SVM	178/226		/	10-CV	83.91%	https://github.com/hangslab/cancerlectins
Phage virion protein	SVM-PVPData[86]	2020	SVM	99/208	30/60	/	JKT	89.58%	http://www.thegleelab.org/PVP-SVM/SVM-PVPData.html
							IDT	79.80%	
Cell wall lytic enzymes	CWLy-RF[87]	2021	RF	68/307		/	10-CV	**96.09%**	/
	CWLy-SVM[88]	2020	SVM			/	JKT	95.50%	http://server.malab.cn/CWLy-SVM/index.jsp
	Jing's method[89]	2021	SVM	68/307		SMOTE	JKT	99.19%	/
Thermophilic Proteins	Feng's method[90]	2020	SVM	915/793		/	10-CV	**98.02%**	http://www.labio.info/index-1therm.html
	Guo's method[91]	2020	SVM			/	CV	96.02%	/

	Li's method[92]	2019	Voting Algorithm			/	10-CV	93.03%	http://lab.malab.cn/~lijing/thermo_data.html
Major Histocompatibility Complex	ELM-MHC[93]	2019	ELM	D1: 4370/4370<br>D2: 3350/3362	D1: 2342/2342	/	10-CV	D1: 91.66%; D2: 92.224%	http://server.malab.cn/ELM-MHC/
							IDT	D1: 93.17%	
Amyloid Proteins	iAMY-SCM[94]	2021	SCM	165/382(P/N=1//)<br>(80%TAD,20%TSD)		/	10-CV	89.50%	http://camt.pythonanywhere.com/iAMY-SCM
							IDT	**82.70%**	
	PredAmyl-MLP[95]	2020	MLP			/	10-CV	**91.59%**	http://106.12.83.135:8080/amyWeb_Release/index.jsp
	RFAmyloid[96]	2018	RF			/	10-CV	89.19%	http://server.malab.cn/RFAmyloid/
							IDT	89.19%	
Antioxidant proteins	ORS-Pred[97]	2021	RF	253/1552		SMOTE	10-CV	97.00%	http://www.labio.info/optiRaac/
	Yu's method[98]	2020	RF	434/1550		/	8-cv	98.20%	/
	AOPs-SVM[99]	2019	SVM	253/1552		/	JKT	94.20%	http://server.malab.cn/AOPs-SVM/index.jsp
Bioluminescent Proteins	Zhao's method[100]	2021	voting algorithm	BLP_General: 4604/7093;<br>BLP_Archaea: 66/748;<br>BLP_Bacteria: 4362/4919;<br>BLP_Eukaryota: 176/1426;<br>(70%TAD, 30%TSD)		/	10-CV	85.00%	/
							IDT	88.40%	
	iBLP[101]	2021	XGBoost	BLP_General: 7956/7093;<br>BLP_Archaea: 45/748;<br>BLP_Bacteria: 748/4919;<br>BLP_Eukaryota: 70/1426;<br>(70%TAD, 30%TSD)		/	10-CV	85.00%	http://lin-group.cn/server/iBLP/
							IDT	88.40%	

Electron Transport Proteins	FastET[102]	2020	SVM	1299/4559		/	5-CV	98.50%	https://github.com/khucnam/FastET
							IDT	96.82%	
	Ru's method[103]	2019	RF	2678/9630		/	10-CV	84.00%	/
							IDT	86.90%	
RNA-Binding proteins	rBPDL[104]	2021	CNN\LSTM	72226/137003		/	10-CV	Macro_AUC: 0.932; Micro_AUC: 0.966	https://github.com/nmt315320/rBPDL
	RBPro-RF[105]	2020	RF	2780/7093	*Human:* 967/597; *S. cerevisiae:* 354/135; *A. thaliana*: 456/37;	SMOTE	10-CV	97.43%	https://github.com/QUST-AIBBDRC/RBPro-RF/
							IDT	*Human:* 95.63%; *S. cerevisiae:* 88.82%; *A. thaliana:* 92.35%;	
	TriPepSVM[106]	2019	SVM	*Human:*1625/10834; *Salmonella*: 275/1273; *E.Coli*: 460/3404;	*Human:*181/1204; *Salmonella: 31/142; E.Coli:* 52/379;	/	10-CV	*Human:*AUC 0.83; *Salmonella*: AUC 0.86; *E.Coli*: AUC 0.92;	https://github.com/QUST-AIBBDRC/RBPro-RF/
							IDT	/	
Plant pentatricopeptide repeat	Feng's method[107]	2021	SVM	487/9590		/	10-CV	**AUC: 0.966**	/
	MixedPPR[108]	2019	RF			/	10-CV	AUC: 0.9848	http://server.malab.cn/MixedPPR/index.jsp
Sub-Golgi protein	isGP-DRLF[109]	2020	SVM	D3: 101/217; D5: 135/1063;	D4: 19/51	SMOTE	LOO	D3: 92.60%; D5:99.20%	http://isgp-drlf.aibiochem.net/
							IDT	D3-model: 98.40% D5-model: 96.42%	
Type III secretion systems	EP3[110]	2020	SVM	TD1: 283/313; TD2: 379/1112;	TSD1:35/86; TSD2:83/14; TSD3: 108/108; TSD4: 226/913;	SMOTE	IDT	EP3_1_model: TSD1: 96.70%; TSD2: 88.70%; TSD3: 77.30%; TSD4: 89.50%; EP3_2_model: TSD1: 81.80%; TSD2:	http://lab.malab.cn/~lijing/EP3.html

								62.90%; TSD3: 92.20%; TSD4: 83.80%	
Multiple PTMs	MultiLyGAN[111]	2021	CWGAN	S1(Ace): 3114; S2(Glyca): 1399; S3(Malon): 1224; S4(Meth): 1147; S5(Succ): 1645; S6(Sumo): 1174; S7(Ubiq): 3185; (4/5TAD,1/5TSD)		/	10-CV	85.89%	https://github.com/Lab-Xu/MultiLyGAN
							IDT	85.49%	
	MusiteDeep[112]	2020	Deep learning	Phosphoserine/threonine: 135556/ 2803647; Phosphortyrosine: 9427/ 93291; N-linked glycosylation: 90344/511755; O-lined glycosylation: 4216/103771; N6-acetyllysine: 22355/274668; Methylarginine: 4675/99946; Methyllysine: 2781/45524; S-palmitoylation-cysteine: 3812/26573;	Phosphoserine/threonine: 8759/ 230755; Phosphortyrosine: 499/ 5540; N-linked glycosylation: 20522/120384; O-lined glycosylation: 218/16248; N6-acetyllysine: 683/11371; Methylarginine: 269/6859; Methyllysine: 154/2001; S-palmitoylation-cysteine: 151/684; Pyrrolidone-carboxylic-acid:	/	10-CV	/	https://www.musite.net/
							IDT	Phosphoserine/threonine: AUC 0.896; Phosphotyrosine: AUC 0.958; N-linked glycosylation: AUC 0.993; O-lined glycosylation: AUC 0.943; N6-acetyllysine: AUC 0.978; Methylarginine: AUC 0.941; Methyllysine: AUC 0.951; S-palmitoylation-cysteine: AUC 0.961; Pyrrolidone-carboxylic-acid: AUC 0.979; Ubiquitination: AUC 0.804; SUMOylation: AUC 0.990; Hydroxylysine: AUC 0.982; Hydroxyproline: AUC 0.732;	

				Pyrrolidone-carboxylic-acid: 1394/10528; Ubiquitination: 3707/49963; SUMOylation: 1225/23932; Hydroxylysine: 356/2650; Hydroxyproline: 2773/11761;	230/ 8918; Ubiquitination: 514/6621; SUMOylation: 65/ 1310; Hydroxylysine: 9/37; Hydroxyproline: 422/814;				
Acetylation	DNNAce[113]	2020	DNN	Archaea: 193/193; B.subtilis:1040/1040; C.glutamicum:1021/1021; E.amylovora:95/95; E.coli:1919/1919; G.kaustophilus:189/189; M.tuberculosis:866/866; S.typhimuricum:174/174; V.parahemolvticus: 1065/1065;	Archaea:21/21; B.subtilis:115/115; C.glutamicum:113/ 113; E.amylovora:10/10; E.coli:213/213; G.kaustophilus:21/ 21; M.tuberculosis:96/ 96; S.typhimuricum:19 /19; V.parahemolvticus: 118/118;	/	10-CV	Archaea: 84.74%; B.*subtilis:* 73.89%; C.*glutamicum:* 75.38%; E.*amylovora:* 96.89%; E.*coli:* 63.08%; G.*kaustophilus:* 89.15%; M.*tuberculosis:* 76.62%; S.*typhimuricum:* 90.51%; V.*parahemolvticus:* 75.46%;	https://github.com/QUST-AIBBDRC/DNNAce/
							IDT	Archaea: 90.00%; B.*subtilis:* 98.26%; C.*glutamicum:* 92.88%; E.*amylovora:* 90.00%; E.*coli:* 86.18%; G.*kaustophilus:* 97.50%; M.*tuberculosis:* 96.44%;	

								S.*typhimuricum:* 95.00%;<br>V.*parahemolvticus:* 94.02%;	
	iAcetyp[114]	2019	RF	725/2715		/	5-CV	77.10%	http://www.jci-bioinfo.cn/iAcetyP
	PAPred[115]	2019	SVM	E.*coli:* 6592/15060;<br>C.*glutamicum:* 1052/6129;<br>M.*tuberculosis:* 865/5167;<br>B.*subtilis:* 1571/12173;<br>S.*typhimurium:* 198/2477;<br>G.*kaustophilus:* 206/1812;	E.*coli:* 361/1384;<br>C.*glutamicum:* 83/830;<br>M.*tuberculosis:* 68/576;<br>B.*subtilis:* 125/1165;<br>S.*typhimurium:* 10/217;<br>G.*kaustophilus:* 17/192;	/	10-CV	**E.*coli:* 77.20%; C.*glutamicum:*75.60%;M.*tuberculosis:* 78.30%;B.*subtilis:* 71.90%; S.*typhimurium:* 82.10%;G.*kaustophilus:* 80.70%;**	http://computbiol.ncu.edu.cn/PAPred#
							IDT	**E.*coli:* 85.10%; C.*glutamicum:* 79.30%;M.*tuberculosis:* 82.70%;B.*subtilis:* 83.10%; S.*typhimurium:* 79.50%;G.*kaustophilus:* 80.90%;**	
	ProAcePred[116]	2018	SVM	Archaea: 193/1590;<br>B.subtilis:1040/5772;<br>C.glutamicum:1021/4333;<br>E.amylovora:95/718;<br>E.coli:1919/1919;<br>G.kaustophilus:189/1025;<br>M.tuberculosis:866/3926;	Archaea:21/176;<br>B.subtilis:115/641;<br>C.glutamicum:113/481;<br>E.amylovora:10/80;<br>E.coli:213/213;<br>G.kaustophilus:21/114;<br>M.tuberculosis:96/436;<br>S.typhimuricum:19/163;	/	10-CV	Archaea: 90.00%;<br>B.subtilis: 79.60%;<br>C.glutamicum:80.00%;<br>E.amylovora: 98.30%;<br>E.coli: 69.00%;<br>G.kaustophilus: 89.70%;<br>M.tuberculosis: 83.40%;<br>S.typhimuricum: 87.40%;<br>V.parahemolvticus: 80.20%;	http://computbiol.ncu.edu.cn/ProAcePred#
							IDT	Archaea: 81.00%;<br>B.subtilis: 95.20%;<br>C.glutamicum:87.20%;	

				S.typhimuricum:174/1467;<br>V.parahemolvticus: 1065/5938;	V.parahemolvticus: 118/659;			E.amylovora: 90.00%;<br>E.coli: 89.90%;<br>G.kaustophilus: 88.10%;<br>M.tuberculosis: 88.00%;<br>S.typhimuricum: 81.60%;<br>V.parahemolvticus: 86.90%;	
	DeepAcet[117]	2019	MLP	12886/12886	3221/3221	/	10-CV	84.95%	https://github.com/Sunmile/DeepAcet
							IDT	84.87%	
Hydroxylation	iHyd-LysSite (EPSV)[118]	2020	NN\RF\SVM	185/497		/	JKT	97.24%	/
	iHyd-PseCp[119]	2016	RF	HyP sites: 851/3505<br>HyL sites: 142/980		/	JKT	HyP sites: 96.58%<br>HyL sites: 97.08%	http://www.jci-bioinfo.cn/iHyd-PseCp
Malonylation	Mal-Prec[120]	2020	SVM	1735/1735 (80%TAD, 20%TSD)		/	5-CV	91.24%	https://github.com/flyinsky6/Mal-Prec
							IDT	90.65%	
	Kmalo[121]	2020	CNN\RF\SVM	*Mammalian:*5006/76264<br>*Plant:* 196/2394	*Mammalian:* 1252/19066<br>*Plant:* 82/1195	/	10-CV	*Mammalian:* 76.40%<br>*Plant:* 66.00%	https://fdblab.csie.ncu.edu.tw/kmalo/home.html
							IDT	*Mammalian:* 86.60%<br>*Plant:* 69.10%	
	KMAL-SP[122]	2019	Ensemble learning	*H. sapiens:*3585/3585;<br>*M.musculus*:2606/2606;<br>*E. coli*:1453/1453;	*H. sapiens:*300/300;<br>*M.musculus:*300/300;<br>*E. coli*:100/100;	/	10-CV	*H. sapiens:* 83.50%;<br>*M. musculus:* 82.50%;<br>*E. coli*: 80.10%;	https://kmalsp.erc.monash.edu/
							IDT	*H. sapiens:*86.00%;<br>*M. musculus:* 83.30%;<br>*E. coli*: 84.50%;	
Methylation	Hou's method[123]	2020	RF	Single-methylarginine: 1465<br>double-methylarginine: 474		/	10-CV	Single: 82.1%; Double: 82.5%	https://github.com/houruiyan/Arginine-methylation-prediction-with-CTD-

				Negative samples: 39980					features
	DeepRMethylSite[124]	2020	CNN\LSTM	8344/244600	2085/61150	Undersampling	5-CV	76.00%	https://github.com/dukkakc/DeepRMethylSite
							IDT	75.00%	
	MePred-RF[125]	2017	RF	*Methylation site R*<br>D1: 185/185; D2: 185/185; D3: 185/185; D4: 185/<br>185; D5: 185/185; D6:185/186; D7: 185/185;<br>*Methylation site K*<br>D1: 226/217; D2: 226/217; D3: 226/<br>217; D4: 226/217; D5: 226/218; D6: 226/217; D7: 226/217;		/	JKT	**Methylation site R**<br>D1: 80.3%; D2: 80.3%; D3: 78.4%; D4: 80.8%; D5: 82.2%; D6: 82.7%; D7: 80.5%; Average: 80.7%;<br>**Methylation site K**<br>D1: 67.7%; D2: 68.4%; D3: 72.7%; D4: 70.0%; D5: 69.0%; D6: 69.3%; D7: 72.5%; Average: 69.9%;	http://server.malab.cn/MePred-RF
Palmitoylation	GPS-Palm[126]	2020	CNN	3089/18992		/	10-CV	*Human:* AUC 0.900<br>*Mouse:* AUC 0.897	http://gpspalm.biocuckoo.cn/
	SPalmitoylC-PseAAC[127]	2019	ANN	436/500		/	Self-consistency testing	99.79%	http://www.biopred.org/palm
							10-CV	97.22%	
Phosphorylation	DeepPPSite[128]	2021	Deep learning	S: 4316/4316;<br>T: 1551/1551;<br>Y: 553/553;	S:2773/17118;<br>T: 941/6258;<br>Y: 210/1296;	/	10-CV	S: 80.38%; T: 80.01%; Y: 77.76%;	https://github.com/saeed344/DeepPPSite
							IDT	S: 78.91%; T: 84.81%; Y: 82.73%;	
	DeepIPs[129]	2021	CNN\LSTM	S/T: 4308/4308<br>Y: 81/81	S/T:1079/1079<br>Y: 21/21	/	5-CV	S/T: 80.45%; Y: 75.22%	http://lin-group.cn/server/DeepIPs/
							IDT	S/T:80.63%; Y: 83.33%	
	DeepPSP[130]	2020	DNN	S/T:<br>165787/879507<br>Y: 28965/134997	S/T: 18588/102113<br>Y: 3248/14504	/	/	S/T: AUC 0.82; Y: AUC 0.73;	https://github.com/gankLei-X/DeepPSP
							IDT	NA	

	iPhoPred[131]	2019	SVM	SerD: 300/300; TyrD: 110/110; ThrD: 100/100;		/	JKT	SerD: AUC 0.904<br>TyrD: AUC 0.992<br>ThrD: AUC 0.990	http://lin-group.cn/server/iPhoPred/
	PhosPred-RF[132]	2017	RF	S-type: 4316/4316;<br>T-type: 1551/1551;<br>Y-type: 553/553;	S-type: 2273/17618; T-type: 941/6258;<br>Y-type: 296/1210;	/	10-CV	S-type: AUC 0.851<br>T-type: AUC 0.818<br>Y-type: AUC 0.761	http://server.malab.cn/PhosPred-RF#
							IDT	S-type: AUC 0.715<br>T-type: AUC 0.683<br>Y-type: AUC 0.654	
Pupylation	PUP-Fuse[133]	2021	RF	186/186	87/191	/	10-CV	88.40%	http://kurata14.bio.kyutech.ac.jp/PUP-Fuse/
							IDT	82.00%	
	Li's method[134]	2018	SVM	183/2258	29/408	/	10-CV	95.09%	/
							IDT	83.75%	
Succinylation	LSTMCNNsucc[135]	2021	LSTM\CNN	6512/6512	1479/16457	/	10-CV	79.90%	http://8.129.111.5/
							IDT	NA	
	CNN-SuccSite[136]	2019	CNN	3216/16412	218/2621	/	10-CV	85.68%	http://csb.cse.yzu.edu.tw/CNN-SuccSite/
							IDT	86.79%	
S-nitrosylation	PreSNO[137]	2019	SVM\RF	3383/3383	351/3168	/	5-CV	70.00%	http://kurata14.bio.kyutech.ac.jp/PreSNO/
							IDT	75.20%	
	Li's method[138]	2019	SVM	731/810	124/221	/	5-CV	83.11%	/
							IDT	73.17%	
	DeepNitro[139]	2018	DNN	Tyrosine nitration: 1210/8043;<br>Tryptophan nitration: 66/155;<br>S-nitrosylation:	Tyrosine nitration: 189/1182;<br>S-nitrosylation: 485/4947;	/	10-CV	Tyrosine nitration: AUC 0.65;<br>Tryptophan nitration: AUC 0.80;<br>S-nitrosylation: AUC 0.70;	http://deepnitro.renlab.org
							IDT	Tyrosine nitration: AUC 0.6879;<br>Tryptophan nitration: AUC 0.8428;	

				3409/17453;				S-nitrosylation: AUC 0.70;	
Tyrosine sulfation	iSulfoTyr-PseAAC[140]	2019	Neural network	200/420	80/80	/	10-CV	94.20%	/
							IDT	85.63%	
Ubiquitination	UbiSite-XGBoost[141]	2021	XGBoost	Set1: 150/150;<br>Set2: 3419/3419;<br>Set3: 6118/6118;<br>Set4: 263/4345;<br>Set5: 131/639;<br>Set6: 37/639;	D1: 92/301;<br>D2: 176/475;<br>D3: 96/666;	SMOTE	5-CV	Set1: AUC 0.8258;<br>Set2: AUC 0.7592;<br>Set3: AUC 0.7853;<br>Set4: AUC 0.9777;<br>Set5: AUC 0.9782;<br>Set6: AUC 0.9860;	https://github.com/QUST-AIBBDRC/UbiSite-XGBoost/
							IDT	D1: 78.09%; D2: 74.19%; D3: 87.13%;	
	CNNAthUbi[142]	2021	CNN	2043/6130	511/1533	/	5-CV	85.38%	https://github.com/nongdaxiaofeng/CNNAthUbi
							IDT	85.36%	
	DeepTL-Ubi[143]	2021	transfer learning	*H.sapiens:* 31162/31162;<br>*M.musculus:* 7746/7748;<br>*S.cerevisiae:* 3506/ 3506;<br>*R.norvegicus:* 1226/1226;<br>*A.nidulans:* 2299/2299;<br>*A.thaliana:* 586/ 587;<br>*T.gondii:* 424/424;<br>*O.sativa:* 308/308;<br>(90%TAD, 10%TSD)		/	/	*H.sapiens:*57.80%;<br>*M.musculus:* 60.40%;<br>*R.norvegicus:* 57.80%;<br>*S.cerevisiae:*59.30%;<br>*A. thaliana:* 52.50%;<br>*O. sativa:* 50.00%;<br>*T.gondii:* 53.80%;<br>*A. nidulans:* 63.60%;	https://github.com/USTC-HIlab/DeepTL-Ubi
							IDT	*H.sapiens*: AUC 0.753;<br>*M.musculus*: AUC 0.789;<br>*R.norvegicus*: AUC 0.72; *S.cerevisiae*: AUC 0.772; *T.gondii*: AUC 0.824;<br>*A.thaliana*: AUC 0.814;	

	UbiSitePred[144]	2019	SVM	Set1: 150/150; Set2: 3418/3418; Set3: 6117/6117;		/	5-CV	Set1: 98.33%; Set2: 81.12%; Set3: 76.90%	https://github.com/QUST-AIBBDRC/UbiSitePred/
Carbonylation	CarSite‑II[145]	2021	SVM	K: 618/26995; P: 162/22418; R: 204/22849; T: 191/24271;	K: 117/7439; P: 16/5318; R: 54/5966; T: 191/24271;	SMOTE-KSU	10-CV	K: 82.73%; P: 82.72%; R: 83.16%; T: 85.37%;	http://47.100.136.41:8081/
							IDT	K: 98.21%; P: 97.92%; R: 97.96%; T: 98.94%;	
	iCarPS[146]	2021	RF	K: 266/1802; P: 114/716; R: 119/754; T: 116/702;	K: 34/147; P: 12/76; R: 17/93; T: 5/30;	/	10-CV	K: AUC 0.789; P: AUC 0.814; R AUC 0.726; T: AUC 0.790;	http://lin-group.cn/server/iCarPS/
							IDT	K: AUC 0.756; P: AUC 0.752; R: AUC 0.6495; T: AUC 0.8400;	
	iCar-PseCp[147]	2020	RF	K: 300/1949; P: 126/792; R: 136/847; T: 121/732;		/	10-CV	K: 84.43%; P: 86.79%; R: 84.23%; T: 86.17%;	http://www.jci-bioinfo.cn/iCar-PseCp
Glutarylation	iGlu_AdaBoost[148]	2021	AdaBoost	400/1703	44/203	SMOTE-Tomek	10-CV	79.89%	/
							IDT	72.07%	
Glycosylation	NIonPred[149]	2021	transfer learning	495/5018	103/1019	/	5-CV	93.40%	https://github.com/khucnam/NIonPred
							IDT	92.90%	
	N-GlyDE[150]	2019	SVM	First_stage: 629/5566;	167/280	/	10-CV	73.30%	http://bioapp.iis.sinica.edu.tw/N-GlyDE/
							IDT	74.00%	

				Second-stage: 2050/1030					
S-sulfenylation	Sulf-DNN[151]	2021	DNN	900/6856	145/268	/	10-CV	79.90%	https://github.com/khanhlee/fastSulf-DNN
							IDT	77.09%	
	SVM-SulfoSite[152]	2018	SVM			/	10-CV	89.00%	https://github.com/HussamAlbarakati/SVM-Sulfosite
							IDT	74.00%	
SUMOylation	mUSP[153]	2020	RF	3363/123131		/	10-CV	AUC 0.8472	http://bioinfo.ncu.edu.cn/mUSP/index.html#
**(II) Peptide**									
Anticancer peptides	iACP-DRLF[154]	2021	LightGBM	Main Dataset: P-861/ N-861<br>Alternate Dataset: P-970/ N-970		/	5-CV	**Main Dataset: 79.1%**<br>**Alternate Dataset: 94.5%**	http://public.aibiochem.net/iACP-DRLF/
							IDT	**Main Dataset: 77.5%**<br>**Alternate Dataset: 93.0%**	
	AntiCP 2.0[155]	2021	ETree			/	5-CV	Main Dataset: 75.29%<br>Alternate Dataset: 90.10%	https://webs.iiitd.edu.in/raghava/anticp2/
							IDT	Main Dataset: 75.43%<br>Alternate Dataset: 92.01%	
	ACPred-Fuse[156]	2020	RF	125/125	82/2628	/	10-CV	82.40%	http://server.malab.cn/ACPred-Fuse
							IDT	89.00%	
	ACPred-FL[157]	2018	SVM	250/250	970/970	/	5-CV	91.40%	http://server.malab.cn/ACPred-FL/
							IDT	88.40%	
Anti-hypertensive peptides	Rauf's method[158]	2021	CNN\SVM	913/913	386/386	/	10-CV	**95.00%**	/
							IDT	**88.90%**	
	mAHTPred[159]	2019	ERT			/	10-CV	84.80%	http://thegleelab.org/mAHTPred/
							IDT	88.30%	

Anti-Tubercular Peptides	AtbPpred[160]	2019	ERT	AntiTb_MD: 199/199<br>AntiTb_RD: 199/199	AntiTb_MD: 47/47<br>AntiTb_RD: 47/47	/	10-CV	AntiTb_MD: 84.90% AntiTb_RD: 91.70%	http://thegleelab.org/AtbPpred/
							IDT	AntiTb_MD: 89.40% AntiTb_RD: 85.10%	
Therapeutic peptides	ITP-Pred[161]	2021	CNN\BiLSTM	QSP400: 400/400<br>CPP740: 740/740	QSP400: 40/40<br>CPP740: 92/92	/	5-CV	QSP400:87.00%<br>CPP740: 87.30%	/
							IDT	QSP400: 95.10%<br>CPP740: 97.50%	
	PPTPP[162]	2020	RF	AAP: 107/107;<br>ABP: 800/800;<br>ACP: 250/250;<br>AIP: 1258/1887;<br>AVP: 544/407;<br>CPP: 370/370;<br>QSP: 200/200;<br>SBP: 80/80;	**Main:**<br>AAP: 28/28;<br>ABP: 199/199;<br>ACP: 82/82;<br>AIP: 420/629;<br>AVP: 60/54;<br>CPP: 92/92;<br>QSP: 20/20;<br>SBP: 24/20;<br>**Alternative:**<br>AAP: 28/2000;<br>ABP: 199/2000;<br>ACP: 82/2000;<br>AIP: 420/2000;<br>AVP: 60/2000;<br>CPP: 92/ 2000;<br>QSP: 20/2000;<br>SBP: 24/2000;	/	10-CV	**AAP: AUC 0.871;**<br>**ABP: AUC 0.977;**<br>**ACP: AUC 0.927;**<br>**AIP: AUC 0.772;**<br>**AVP: AUC 0.930;**<br>**CPP: AUC 0.975;**<br>**QSP: AUC 0.972;**<br>**SBP: AUC 0.865;**	https://github.com/YPZ858/PPTPP
							IDT	**Main AUC:**<br>**AAP: 0.770; ABP: 0.988; ACP: 0.883; AIP: 0.720; AVP: 0.946; CPP: 0.965; QSP: 0.944; SBP: 0.740;**	
	PEPred-Suite[163]	2019	RF			/	10-CV	AAP: AUC 0.874; ABP: AUC 0.976; ACP: AUC 0.950; AIP: AUC 0.778; AVP: AUC 0.924;<br>CPP: AUC 0.972; QSP: AUC 0.968; SBP: AUC 0.813;	http://server.malab.cn/PEPred-Suite
							IDT	**Main AUC:**	

								AAP: 0.804; ABP: 0.976; ACP: 0.949; AIP: 0.751; AVP: 0.949; CPP: 0.952; QSP: 0.960; SBP: 0.679; **Alternative AUC:** AAP: 0.774; ABP: 0.969; ACP: 0.638; AIP: 0.638; AVP: 0.945; CPP: 0.878; QSP: 0.897; SBP: 0.796;	
Toxic peptides	ATSE[164]	2021	Deep learning	1932/1932 (85%TAD, 15%TSD)		/	10-CV	95.20%	http://server.malab.cn/ATSE
							IDT	NA	
Cell-penetrating Peptides	TargetCPP[165]	2020	GBDT	462/462	111/34	/	JKT	93.45%	/
							IDT	88.28%	
	StackCPPred[166]	2020	SVM	CPP924: 462/462; CPPsite3: 187/187		/	JKT	**CPP924: 94.50%** **CPPsite3: 78.3%**	https://github.com/Excelsior511/StackCPPred
	CPPred-FL[167]	2018	RF			/	10-CV	CPP924: 92.10%	http://server.malab.cn/CPPred-FL
	SkipCPP-Pred[168]	2018	RF			/	JKT	CPP924: 90.60%	http://server.malab.cn/SkipCPP-Pred/Index.html
	CPPred-RF[169]	2017	RF			/	JKT	CPP924: 91.60%; CPPsite3: 71.10%	http://server.malab.cn/CPPred-RF
Hemolytic peptide	HLPpred-Fuse[170]	2020	ERT	D1: 433/666; D2: 433/106; D3: 671/168;	D1: 423/1999; D2: 352/92; D3: 559/147;	/	10-CV	D1: 98.40%; D2: 81.30%; D3: 82.90%	http://thegleelab.org/HLPpred-Fuse
							IDT	Dataset_1: BACC: 90.50%; Dataset_2: Acc:80.80%; Dataset_3: BACC: 79.20%;	
	HemoPlmod[171]	2020	RF	466/466	117/117	/	5-CV	78.30%	http://webs.iiitd.edu.in/raghava/hemopimod/
							IDT	78.29%	
	HAPPENN[17	2020	ANN	1543/2195		/	10-CV	85.66%±1.93	https://research.timmons.eu/happenn

	2]								
Bitter Peptide	iBitter-Fuse[173]	2021	SVM	256/256	64/64	/	10-CV	**91.80%**	http://camt.pythonanywhere.com/iBitter-Fuse
							IDT	**93.00%**	
	BERT4Bitter[174]	2021	BERT			/	10-CV	81.60%	http://pmlab.pythonanywhere.com/BERT4Bitter
							IDT	92.20%	
	iBitter-SCM[175]	2020	SCM			/	10-CV	87.11%	http://camt.pythonanywhere.com/iBitter-SCM
							IDT	84.38%	

Note: # URL is not available. P: positive samples; N: negative samples; TAD: Training dataset; TSD: Testing dataset; 5-CV: 5-fold cross validation; 10-CV: 10-fold cross validation; IDT: Independent testing; JKT: jackknife test. SMOTE: Synthetic Minority Oversampling Technique; SMOTE-KSU: K-means similarity based under sampling and the synthetic minority oversampling technique; SMOTE-Tomek: Synthetic Minority Over-sampling Technique (SMOTE) and the under sampling method Tomek; GBDT: gradient-boosting decision tree; SVM: support vector machine; RF: random forest; DNN: deep neural networks; CNN: convolutional neural network; ERT: extremely randomized tree; ELM: extreme learning machine; XGBoost: extreme gradient boosting; LightGBM: light gradient boosting; Etree: extra trees; ANN: artificial neural network; SCM: scoring card method; GBM: gradient boosting machine; MLP: multi-layer perceptron; LSTM: long short-term memory; CWGAN: conditional Wasserstein generative adversarial network; HMM: Hidden Markov model; BiLSTM: directional Long Short-Term Memory;

REFERENCES

1. Lim, D.Y., et al., *iEnhancer-RF: Identifying enhancers and their strength by enhanced feature representation using random forest.* Chemometrics and Intelligent Laboratory Systems, 2021. **212**.
2. Cai, L., et al., *iEnhancer-XG: interpretable sequence-based enhancers and their strength predictor.* Bioinformatics, 2021. **37**(8): p. 1060-1067.
3. Liu, B., et al., *iEnhancer-EL: identifying enhancers and their strength with ensemble learning approach.* Bioinformatics, 2018. **34**(22): p. 3835-3842.
4. Jia, C. and W. He, *EnhancerPred: a predictor for discovering enhancers based on the combination and selection of multiple features.* Scientific Reports, 2016. **6**.
5. Liu, B., et al., *iEnhancer-2L: a two-layer predictor for identifying enhancers and their strength by pseudo k-tuple nucleotide composition.* Bioinformatics, 2016. **32**(3): p. 362-9.
6. Xiao, X., et al., *iPSW(2L)-PseKNC: A two-layer predictor for identifying promoters and their strength by hybrid features via pseudo K-tuple nucleotide composition.* Genomics, 2019. **111**(6): p. 1785-1793.

7. Lyu, Y., et al., *iPro2L-PSTKNC: A Two-Layer Predictor for Discovering Various Types of Promoters by Position Specific of Nucleotide Composition.* Ieee Journal of Biomedical and Health Informatics, 2021. **25**(6): p. 2329-2337.
8. Liu, B., et al., *iPromoter-2L: a two-layer predictor for identifying promoters and their types by multi-window-based PseKNC.* Bioinformatics, 2018. **34**(1): p. 33-40.
9. Jia, C., Q. Yang, and Q. Zou, *NucPosPred: Predicting species-specific genomic nucleosome positioning via four different modes of general PseKNC.* Journal of Theoretical Biology, 2018. **450**: p. 15-21.
10. Li, J., et al., *DeepATT: a hybrid category attention neural network for identifying functional effects of DNA sequences.* Briefings in bioinformatics, 2021. **22**(3).
11. Khanal, J., et al., *Identifying DNA N4-methylcytosine sites in the rosaceae genome with a deep learning model relying on distributed feature representation.* Comput Struct Biotechnol J, 2021. **19**: p. 1612-1619.
12. Lv, Z., et al., *Escherichia Coli DNA N-4-Methycytosine Site Prediction Accuracy Improved by Light Gradient Boosting Machine Feature Selection Technology.* IEEE Access, 2020. **8**: p. 14851-14859.
13. Zeng, R. and M. Liao, *Developing a Multi-Layer Deep Learning Based Predictive Model to Identify DNA N4-Methylcytosine Modifications.* Frontiers in bioengineering and biotechnology, 2020. **8**: p. 274-274.
14. Wei, L., et al., *Iterative feature representations improve N4-methylcytosine site prediction.* Bioinformatics, 2019. **35**(23): p. 4930-4937.
15. Wei, L., et al., *Exploring sequence-based features for the improved prediction of DNA N4-methylcytosine sites in multiple species.* Bioinformatics, 2019. **35**(8): p. 1326-1333.
16. He, W., C. Jia, and Q. Zou, *4mCPred: machine learning methods for DNA N4-methylcytosine sites prediction.* Bioinformatics, 2019. **35**(4): p. 593-601.
17. Manavalan, B., et al., *Meta-4mCpred: A Sequence-Based Meta-Predictor for Accurate DNA 4mC Site Prediction Using Effective Feature Representation.* Mol Ther Nucleic Acids, 2019. **16**: p. 733-744.
18. Manavalan, B., et al., *4mCpred-EL: An Ensemble Learning Framework for Identification of DNA N(4)-methylcytosine Sites in the Mouse Genome.* Cells, 2019. **8**(11).
19. Liu, M.Y., et al., *MGF6mARice: prediction of DNA N6-methyladenine sites in rice by exploiting molecular graph feature and residual block.* Briefings in Bioinformatics, 2022. **23**(3).
20. Li, Z.T., et al., *Deep6mA: A deep learning framework for exploring similar patterns in DNA N6-methyladenine sites across different species.* Plos Computational Biology, 2021. **17**(2).
21. Lv, Z., et al., *A Convolutional Neural Network Using Dinucleotide One-hot Encoder for identifying DNA N6-Methyladenine Sites in the Rice Genome.* Neurocomputing, 2021. **422**: p. 214-221.
22. Huang, Q., et al., *6mA-RicePred: A Method for Identifying DNA N (6)-Methyladenine Sites in the Rice Genome Based on Feature Fusion.* Front Plant Sci, 2020. **11**: p.

4.
23. Wahab, A., et al., *iIM-CNN: Intelligent Identifier of 6mA Sites on Different Species by Using Convolution Neural Network.* Ieee Access, 2019. **7**: p. 178577-178583.
24. Liu, Z., et al., *csDMA: an improved bioinformatics tool for identifying DNA 6 mA modifications via Chou's 5-step rule.* Scientific Reports, 2019. **9**.
25. Zhang, L., X. Xiao, and Z.C. Xu, *iPromoter-5mC: A Novel Fusion Decision Predictor for the Identification of 5-Methylcytosine Sites in Genome-Wide DNA Promoters.* Front Cell Dev Biol, 2020. **8**: p. 614.
26. Liu, Z., et al., *iDNA-Methyl: Identifying DNA methylation sites via pseudo trinucleotide composition.* Analytical Biochemistry, 2015. **474**: p. 69-77.
27. Li, J., et al., *SubLocEP: a novel ensemble predictor of subcellular localization of eukaryotic mRNA based on machine learning.* Brief Bioinform, 2021. **22**(5).
28. Meng, J., et al., *PlncRNA-HDeep: plant long noncoding RNA prediction using hybrid deep learning based on two encoding styles.* Bmc Bioinformatics, 2021. **22**(SUPPL 3).
29. Liu, S., et al., *PredLnc-GFStack: A Global Sequence Feature Based on a Stacked Ensemble Learning Method for Predicting lncRNAs from Transcripts.* Genes, 2019. **10**(9).
30. Wang, L., et al., *ncRFP: A Novel end-to-end Method for Non-Coding RNAs Family Prediction Based on Deep Learning.* Ieee-Acm Transactions on Computational Biology and Bioinformatics, 2021. **18**(2): p. 784-789.
31. Chantsalnyam, T., et al., *ncRDense: A novel computational approach for classification of non-coding RNA family by deep learning.* Genomics, 2021. **113**(5): p. 3030-3038.
32. Chantsalnyam, T., et al., *ncRDeep: Non-coding RNA classification with convolutional neural network.* Computational Biology and Chemistry, 2020. **88**.
33. Meher, P.K., et al., *ASRmiRNA: Abiotic Stress-Responsive miRNA Prediction in Plants by Using Machine Learning Algorithms with Pseudo K-Tuple Nucleotide Compositional Features.* International Journal of Molecular Sciences, 2022. **23**(3).
34. Zheng, X., et al., *Deep neural networks for human microRNA precursor detection.* Bmc Bioinformatics, 2020. **21**(1).
35. Niu, M., et al., *CirRNAPL: A web server for the identification of circRNA based on extreme learning machine.* Computational and Structural Biotechnology Journal, 2020. **18**: p. 834-842.
36. Zou, Q., et al., *Improving tRNAscan-SE Annotation Results via Ensemble Classifiers.* Molecular Informatics, 2015. **34**(11-12): p. 761-770.
37. Ali, S.D., et al., *Identification of Functional piRNAs Using a Convolutional Neural Network.* IEEE/ACM transactions on computational biology and bioinformatics, 2020. **PP**.
38. Liu, B., F. Yang, and K.-C. Chou, *2L-piRNA: A Two-Layer Ensemble Classifier for Identifying Piwi-Interacting RNAs and Their Function.* Molecular Therapy-Nucleic Acids, 2017. **7**: p. 267-277.

39. Niu, M. and Q. Zou, *SgRNA-RF: identification of SgRNA on-target activity with imbalanced datasets.* IEEE/ACM Transactions on Computational Biology and Bioinformatics, 2021: p. 1-1.
40. Song, Z., et al., *Attention-based multi-label neural networks for integrated prediction and interpretation of twelve widely occurring RNA modifications.* Nature Communications, 2021. **12**(1).
41. Liu, K. and W. Chen, *iMRM: a platform for simultaneously identifying multiple kinds of RNA modifications.* Bioinformatics, 2020. **36**(11): p. 3336-3342.
42. Lv, Z., et al., *RF-PseU: A Random Forest Predictor for RNA Pseudouridine Sites.* Front Bioeng Biotechnol, 2020. **8**: p. 134.
43. Tahir, M., H. Tayara, and K.T. Chong, *iPseU-CNN: Identifying RNA Pseudouridine Sites Using Convolutional Neural Networks.* Mol Ther Nucleic Acids, 2019. **16**: p. 463-470.
44. Dou, L., et al., *Is There Any Sequence Feature in the RNA Pseudouridine Modification Prediction Problem?* Mol Ther Nucleic Acids, 2020. **19**: p. 293-303.
45. Dou, L., et al., *Prediction of m5C Modifications in RNA Sequences by Combining Multiple Sequence Features.* Mol Ther Nucleic Acids, 2020. **21**: p. 332-342.
46. Lv, H., et al., *Evaluation of different computational methods on 5-methylcytosine sites identification.* Brief Bioinform, 2020. **21**(3): p. 982-995.
47. Dou, L., et al., *iRNA-m5C_NB: A Novel Predictor to Identify RNA 5-Methylcytosine Sites Based on the Naive Bayes Classifier.* Ieee Access, 2020. **8**: p. 84906-84917.
48. Ali, S.D., et al., *Prediction of RNA 5-Hydroxymethylcytosine Modifications Using Deep Learning.* IEEE Access, 2021. **9**: p. 8491-8496.
49. Liu, Y., et al., *iRNA5hmC: The First Predictor to Identify RNA 5-Hydroxymethylcytosine Modifications Using Machine Learning.* Front Bioeng Biotechnol, 2020. **8**: p. 227.
50. Zhang, L., et al., *DNN-m6A: A Cross-Species Method for Identifying RNA N6-Methyladenosine Sites Based on Deep Neural Network with Multi-Information Fusion.* Genes, 2021. **12**(3): p. 354.
51. Dao, F.-Y., et al., *Computational identification of N6-methyladenosine sites in multiple tissues of mammals.* Computational and Structural Biotechnology Journal, 2020. **18**: p. 1084-1091.
52. Li, J., et al., *HSM6AP: a high-precision predictor for the Homo sapiens N6-methyladenosine (m^6 A) based on multiple weights and feature stitching.* RNA Biol, 2021: p. 1-11.
53. Chen, K., et al., *WHISTLE: a high-accuracy map of the human N6-methyladenosine (m6A) epitranscriptome predicted using a machine learning approach.* Nucleic Acids Res, 2019. **47**(7): p. e41.
54. Mahmoudi, O., A. Wahab, and K.T. Chong, *iMethyl-Deep: N6 Methyladenosine Identification of Yeast Genome with Automatic Feature Extraction Technique by Using Deep Learning Algorithm.* Genes (Basel), 2020. **11**(5).
55. Wei, L., et al., *Integration of deep feature representations and handcrafted features to improve the prediction of N-6-methyladenosine sites.* Neurocomputing, 2019.

324: p. 3-9.
56. Chen, W., P. Xing, and Q. Zou, *Detecting N(6)-methyladenosine sites from RNA transcriptomes using ensemble Support Vector Machines.* Sci Rep, 2017. **7**: p. 40242.
57. Liu, Z., et al., *HLMethy: a machine learning-based model to identify the hidden labels of m(6)A candidates.* Plant Mol Biol, 2019. **101**(6): p. 575-584.
58. Zou, Q., et al., *Gene2vec: gene subsequence embedding for prediction of mammalian N (6)-methyladenosine sites from mRNA.* Rna, 2019. **25**(2): p. 205-218.
59. Huang, Y., et al., *BERMP: a cross-species classifier for predicting m(6)A sites by integrating a deep learning algorithm and a random forest approach.* Int J Biol Sci, 2018. **14**(12): p. 1669-1677.
60. Ao, C., Q. Zou, and L. Yu, *NmRF: identification of multispecies RNA 2'-O-methylation modification sites from RNA sequences.* Briefings in bioinformatics, 2022. **23**(1).
61. Tahir, M., H. Tayara, and K.T. Chong, *iRNA-PseKNC(2methyl): Identify RNA 2'-O-methylation sites by convolution neural network and Chou's pseudo components.* J Theor Biol, 2019. **465**: p. 1-6.
62. Yang, H., et al., *iRNA-2OM: A Sequence-Based Predictor for Identifying 2'-O-Methylation Sites in Homo sapiens.* J Comput Biol, 2018. **25**(11): p. 1266-1277.
63. Ao, C., Q. Zou, and L. Yu, *RFhy-m2G: Identification of RNA N2-methylguanosine modification sites based on random forest and hybrid features.* Methods, 2021.
64. Dou, L., et al., *Accurate identification of RNA D modification using multiple features.* RNA Biol, 2021: p. 1-11.
65. Zou, H. and Z. Yin, *m7G-DPP: Identifying N7-methylguanosine sites based on dinucleotide physicochemical properties of RNA.* Biophys Chem, 2021. **279**: p. 106697.
66. Dai, C., et al., *Iterative feature representation algorithm to improve the predictive performance of N7-methylguanosine sites.* Brief Bioinform, 2021. **22**(4).
67. Chen, W., et al., *iRNA-m7G: Identifying N(7)-methylguanosine Sites by Fusing Multiple Features.* Mol Ther Nucleic Acids, 2019. **18**: p. 269-274.
68. Liu, X., et al., *m7GPredictor: An improved machine learning-based model for predicting internal m7G modifications using sequence properties.* Anal Biochem, 2020. **609**: p. 113905.
69. Hu, J., et al., *TargetDBP+: Enhancing the Performance of Identifying DNA-Binding Proteins via Weighted Convolutional Features.* J Chem Inf Model, 2021. **61**(1): p. 505-515.
70. Lu, W., et al., *Use Chou's 5-Step Rule to Predict DNA-Binding Proteins with Evolutionary Information.* Biomed Res Int, 2020. **2020**: p. 6984045.
71. Wang, J., et al., *BastionHub: a universal platform for integrating and analyzing substrates secreted by Gram-negative bacteria.* Nucleic Acids Research, 2021. **49**(D1): p. D651-D659.
72. Ding, C., et al., *iT3SE-PX: Identification of Bacterial Type III Secreted Effectors Using PSSM Profiles and XGBoost Feature Selection.* Computational and Mathematical Methods in Medicine, 2021. **2021**.
73. Hui, X., et al., *T3SEpp: an Integrated Prediction Pipeline for Bacterial Type III Secreted Effectors.* Msystems, 2020. **5**(4).

74. Thumuluri, V., et al., *NetSolP: predicting protein solubility in Escherichia coli using language models.* Bioinformatics, 2022. **38**(4): p. 941-946.
75. Hon, J., et al., *SoluProt: prediction of soluble protein expression in Escherichia coli.* Bioinformatics, 2021. **37**(1): p. 23-28.
76. Wang, X., et al., *ASPIRER: a new computational approach for identifying non-classical secreted proteins based on deep learning.* Briefings in Bioinformatics, 2022.
77. Wang, C., et al., *NonClasGP-Pred: robust and efficient prediction of non-classically secreted proteins by integrating subset-specific optimal models of imbalanced data.* Microbial Genomics, 2020. **6**(12).
78. Zhang, Y., et al., *PeNGaRoo, a combined gradient boosting and ensemble learning framework for predicting non-classical secreted proteins.* Bioinformatics, 2020. **36**(3): p. 704-712.
79. Zhang, Y., et al., *T4SEfinder: a bioinformatics tool for genome-scale prediction of bacterial type IV secreted effectors using pre-trained protein language model.* Briefings in bioinformatics, 2022. **23**(1).
80. Han, H., et al., *iT4SE-EP: Accurate Identification of Bacterial Type IV Secreted Effectors by Exploring Evolutionary Features from Two PSI-BLAST Profiles.* Molecules, 2021. **26**(9).
81. Wang, J., et al., *Systematic analysis and prediction of type IV secreted effector proteins by machine learning approaches.* Briefings in Bioinformatics, 2019. **20**(3): p. 931-951.
82. Feng, C., et al., *CRCF: A Method of Identifying Secretory Proteins of Malaria Parasites.* IEEE/ACM Trans Comput Biol Bioinform, 2021. **Pp**.
83. Meng, C., L. Wei, and Q. Zou, *SecProMTB: Support Vector Machine-Based Classifier for Secretory Proteins Using Imbalanced Data Sets Applied to Mycobacterium tuberculosis.* Proteomics, 2019. **19**(17).
84. Tang, F., et al., *The accurate prediction and characterization of cancerlectin by a combined machine learning and GO analysis.* Brief Bioinform, 2021.
85. Qian, L., Y. Wen, and G. Han, *Identification of Cancerlectins Using Support Vector Machines With Fusion of G-Gap Dipeptide.* Front Genet, 2020. **11**: p. 275.
86. Meng, C., et al., *Review and comparative analysis of machine learning-based phage virion protein identification methods.* Biochim Biophys Acta Proteins Proteom, 2020. **1868**(6): p. 140406.
87. Jiao, S., L. Xu, and Y. Ju, *CWLy-RF: A novel approach for identifying cell wall lyases based on random forest classifier.* Genomics, 2021. **113**(5): p. 2919-2924.
88. Meng, C., F. Guo, and Q. Zou, *CWLy-SVM: A support vector machine-based tool for identifying cell wall lytic enzymes.* Comput Biol Chem, 2020. **87**: p. 107304.
89. Jing, X.Y. and F.M. Li, *Predicting Cell Wall Lytic Enzymes Using Combined Features*. Front Bioeng Biotechnol, 2020. **8**: p. 627335.
90. Feng, C., et al., *A Method for Prediction of Thermophilic Protein Based on Reduced Amino Acids and Mixed Features.* Front Bioeng Biotechnol, 2020. **8**: p. 285.
91. Guo, Z., et al., *Discrimination of Thermophilic Proteins and Non-thermophilic Proteins Using Feature Dimension Reduction.* Front Bioeng Biotechnol, 2020. **8**: p. 584807.

92. Li, J., P. Zhu, and Q. Zou. *Prediction of Thermophilic Proteins Using Voting Algorithm*. in *7th International Work-Conference on Bioinformatics and Biomedical Engineering (IWBBIO)*. 2019. Granada, SPAIN.
93. Li, Y., M. Niu, and Q. Zou, *ELM-MHC: An Improved MHC Identification Method with Extreme Learning Machine Algorithm.* J Proteome Res, 2019. **18**(3): p. 1392-1401.
94. Charoenkwan, P., et al., *iAMY-SCM: Improved prediction and analysis of amyloid proteins using a scoring card method with propensity scores of dipeptides.* Genomics, 2021. **113**(1 Pt 2): p. 689-698.
95. Li, Y., et al., *PredAmyl-MLP: Prediction of Amyloid Proteins Using Multilayer Perceptron*. Comput Math Methods Med, 2020. **2020**: p. 8845133.
96. Niu, M., et al., *RFAmyloid: A Web Server for Predicting Amyloid Proteins.* Int J Mol Sci, 2018. **19**(7).
97. Feng, C., et al., *ORS-Pred: An optimized reduced scheme-based identifier for antioxidant proteins.* Proteomics, 2021. **21**(15): p. e2100017.
98. Ao, C., et al., *Prediction of antioxidant proteins using hybrid feature representation method and random forest.* Genomics, 2020. **112**(6): p. 4666-4674.
99. Meng, C., et al., *AOPs-SVM: A Sequence-Based Classifier of Antioxidant Proteins Using a Support Vector Machine.* Front Bioeng Biotechnol, 2019. **7**: p. 224.
100. Zhao, S., et al., *Bioluminescent Proteins Prediction with Voting Strategy.* Current Bioinformatics, 2021. **16**(2): p. 240-251.
101. Zhang, D., et al., *iBLP: An XGBoost-Based Predictor for Identifying Bioluminescent Proteins.* Comput Math Methods Med, 2021. **2021**: p. 6664362.
102. Nguyen, D., et al., *Use Chou's 5-steps rule with different word embedding types to boost performance of electron transport protein prediction model.* IEEE/ACM Trans Comput Biol Bioinform, 2020. **Pp**.
103. Ru, X., L. Li, and Q. Zou, *Incorporating Distance-Based Top-n-gram and Random Forest To Identify Electron Transport Proteins.* J Proteome Res, 2019. **18**(7): p. 2931-2939.
104. Niu, M., et al., *rBPDL:Predicting RNA-Binding Proteins Using Deep Learning.* IEEE J Biomed Health Inform, 2021. **25**(9): p. 3668-3676.
105. Sun, X., et al., *RBPro-RF: Use Chou's 5-steps rule to predict RNA-binding proteins via random forest with elastic net.* Chemometrics and Intelligent Laboratory Systems, 2020. **197**.
106. Bressin, A., et al., *TriPepSVM: de novo prediction of RNA-binding proteins based on short amino acid motifs.* Nucleic Acids Res, 2019. **47**(9): p. 4406-4417.
107. Feng, C., Q. Zou, and D. Wang, *Using a low correlation high orthogonality feature set and machine learning methods to identify plant pentatricopeptide repeat coding gene/protein.* Neurocomputing, 2021. **424**: p. 246-254.
108. Qu, K., et al., *Identifying Plant Pentatricopeptide Repeat Coding Gene/Protein Using Mixed Feature Extraction Methods.* Front Plant Sci, 2018. **9**: p. 1961.
109. Lv, Z., et al., *Identification of Sub-Golgi protein localization by use of deep representation learning features.* Bioinformatics, 2020. **36**(24): p. 5600-9.
110. Li, J., et al., *EP3: an ensemble predictor that accurately identifies type III secreted effectors.* Brief Bioinform, 2021. **22**(2): p. 1918-1928.

111. Yang, Y., et al., *Prediction and analysis of multiple protein lysine modified sites based on conditional wasserstein generative adversarial networks.* BMC bioinformatics, 2021. **22**(1): p. 171-171.
112. Wang, D., et al., *MusiteDeep: a deep-learning based webserver for protein post-translational modification site prediction and visualization.* Nucleic Acids Res, 2020. **48**(W1): p. W140-w146.
113. Yu, B., et al., *DNNAce: Prediction of prokaryote lysine acetylation sites through deep neural networks with multi-information fusion.* Chemometrics and Intelligent Laboratory Systems, 2020. **200**.
114. Qiu, W.R., et al., *Identifying Acetylation Protein by Fusing Its PseAAC and Functional Domain Annotation.* Front Bioeng Biotechnol, 2019. **7**: p. 311.
115. Chen, G., et al., *Prediction and functional analysis of prokaryote lysine acetylation site by incorporating six types of features into Chou's general PseAAC.* J Theor Biol, 2019. **461**: p. 92-101.
116. Chen, G., et al., *ProAcePred: prokaryote lysine acetylation sites prediction based on elastic net feature optimization.* Bioinformatics, 2018. **34**(23): p. 3999-4006.
117. Wu, M., et al., *A deep learning method to more accurately recall known lysine acetylation sites.* BMC Bioinformatics, 2019. **20**(1): p. 49.
118. Mahmood, M.K., et al., *iHyd-LysSite (EPSV): Identifying Hydroxylysine Sites in Protein Using Statistical Formulation by Extracting Enhanced Position and Sequence Variant Feature Technique.* Curr Genomics, 2020. **21**(7): p. 536-545.
119. Qiu, W.R., et al., *iHyd-PseCp: Identify hydroxyproline and hydroxylysine in proteins by incorporating sequence-coupled effects into general PseAAC.* Oncotarget, 2016. **7**(28): p. 44310-44321.
120. Liu, X., et al., *Mal-Prec: computational prediction of protein Malonylation sites via machine learning based feature integration : Malonylation site prediction.* BMC Genomics, 2020. **21**(1): p. 812.
121. Chung, C.R., et al., *Incorporating hybrid models into lysine malonylation sites prediction on mammalian and plant proteins.* Sci Rep, 2020. **10**(1): p. 10541.
122. Zhang, Y., et al., *Computational analysis and prediction of lysine malonylation sites by exploiting informative features in an integrative machine-learning framework.* Brief Bioinform, 2019. **20**(6): p. 2185-2199.
123. Hou, R., et al., *Computational Prediction of Protein Arginine Methylation Based on Composition-Transition-Distribution Features.* ACS Omega, 2020. **5**(42): p. 27470-27479.
124. Chaudhari, M., et al., *DeepRMethylSite: a deep learning based approach for prediction of arginine methylation sites in proteins.* Mol Omics, 2020. **16**(5): p. 448-454.
125. Wei, L., et al., *Fast Prediction of Protein Methylation Sites Using a Sequence-Based Feature Selection Technique.* IEEE/ACM Trans Comput Biol Bioinform, 2019. **16**(4): p. 1264-1273.
126. Ning, W., et al., *GPS-Palm: a deep learning-based graphic presentation system for the prediction of S-palmitoylation sites in proteins.* Brief Bioinform, 2021. **22**(2):

p. 1836-1847.
127. Hussain, W., et al., *SPalmitoylC-PseAAC: A sequence-based model developed via Chou's 5-steps rule and general PseAAC for identifying S-palmitoylation sites in proteins.* Anal Biochem, 2019. **568**: p. 14-23.
128. Ahmed, S., et al., *DeepPPSite: A deep learning-based model for analysis and prediction of phosphorylation sites using efficient sequence information.* Anal Biochem, 2021. **612**: p. 113955.
129. Lv, H., et al., *DeepIPs: comprehensive assessment and computational identification of phosphorylation sites of SARS-CoV-2 infection using a deep learning-based approach.* Brief Bioinform, 2021.
130. Guo, L., et al., *DeepPSP: A Global-Local Information-Based Deep Neural Network for the Prediction of Protein Phosphorylation Sites.* J Proteome Res, 2021. **20**(1): p. 346-356.
131. Li, S.-H., et al., *iPhoPred: A Predictor for Identifying Phosphorylation Sites in Human Protein.* Ieee Access, 2019. **7**: p. 177517-177528.
132. Wei, L., et al., *PhosPred-RF: A Novel Sequence-Based Predictor for Phosphorylation Sites Using Sequential Information Only.* IEEE Trans Nanobioscience, 2017. **16**(4): p. 240-247.
133. Auliah, F.N., et al., *PUP-Fuse: Prediction of Protein Pupylation Sites by Integrating Multiple Sequence Representations.* Int J Mol Sci, 2021. **22**(4).
134. Li, T., et al., *Recognition of Protein Pupylation Sites by Adopting Resampling Approach.* Molecules, 2018. **23**(12).
135. Huang, G., et al., *LSTMCNNsucc: A Bidirectional LSTM and CNN-Based Deep Learning Method for Predicting Lysine Succinylation Sites.* Biomed Res Int, 2021. **2021**: p. 9923112.
136. Huang, K.Y., J.B. Hsu, and T.Y. Lee, *Characterization and Identification of Lysine Succinylation Sites based on Deep Learning Method.* Sci Rep, 2019. **9**(1): p. 16175.
137. Hasan, M.M., et al., *Prediction of S-nitrosylation sites by integrating support vector machines and random forest.* Mol Omics, 2019. **15**(6): p. 451-458.
138. Li, T., et al., *Identification of S-nitrosylation sites based on multiple features combination.* Sci Rep, 2019. **9**(1): p. 3098.
139. Xie, Y., et al., *DeepNitro: Prediction of Protein Nitration and Nitrosylation Sites by Deep Learning.* Genomics Proteomics Bioinformatics, 2018. **16**(4): p. 294-306.
140. Barukab, O., et al., *iSulfoTyr-PseAAC: Identify Tyrosine Sulfation Sites by Incorporating Statistical Moments via Chou's 5-steps Rule and Pseudo Components.* Curr Genomics, 2019. **20**(4): p. 306-320.
141. Liu, Y., et al., *Prediction of protein ubiquitination sites via multi-view features based on eXtreme gradient boosting classifier.* J Mol Graph Model, 2021. **107**: p. 107962.
142. Wang, X., et al., *Computational identification of ubiquitination sites in Arabidopsis thaliana using convolutional neural networks.* Plant Mol Biol, 2021. **105**(6): p. 601-610.

143. Liu, Y., et al., *DeepTL-Ubi: A novel deep transfer learning method for effectively predicting ubiquitination sites of multiple species.* Methods, 2021. **192**: p. 103-111.
144. Cui, X., et al., *UbiSitePred: A novel method for improving the accuracy of ubiquitination sites prediction by using LASSO to select the optimal Chou's pseudo components.* Chemometrics and Intelligent Laboratory Systems, 2019. **184**: p. 28-43.
145. Zuo, Y., et al., *CarSite-II: an integrated classification algorithm for identifying carbonylated sites based on K-means similarity-based undersampling and synthetic minority oversampling techniques.* BMC Bioinformatics, 2021. **22**(1): p. 216.
146. Zhang, D., et al., *iCarPS: a computational tool for identifying protein carbonylation sites by novel encoded features.* Bioinformatics, 2021. **37**(2): p. 171-177.
147. Jia, J., et al., *iCar-PseCp: identify carbonylation sites in proteins by Monte Carlo sampling and incorporating sequence coupled effects into general PseAAC.* Oncotarget, 2016. **7**(23): p. 34558-70.
148. Dou, L., et al., *iGlu_AdaBoost: Identification of Lysine Glutarylation Using the AdaBoost Classifier.* J Proteome Res, 2021. **20**(1): p. 191-201.
149. Nguyen, T.T., et al., *Incorporating a transfer learning technique with amino acid embeddings to efficiently predict N-linked glycosylation sites in ion channels.* Comput Biol Med, 2021. **130**: p. 104212.
150. Pitti, T., et al., *N-GlyDE: a two-stage N-linked glycosylation site prediction incorporating gapped dipeptides and pattern-based encoding.* Sci Rep, 2019. **9**(1): p. 15975.
151. Do, D.T., T.Q.T. Le, and N.Q.K. Le, *Using deep neural networks and biological subwords to detect protein S-sulfenylation sites.* Brief Bioinform, 2021. **22**(3).
152. Al-Barakati, H.J., et al., *SVM-SulfoSite: A support vector machine based predictor for sulfenylation sites.* Sci Rep, 2018. **8**(1): p. 11288.
153. Xu, H.D., et al., *mUSP: a high-accuracy map of the in situ crosstalk of ubiquitylation and SUMOylation proteome predicted via the feature enhancement approach.* Brief Bioinform, 2021. **22**(3).
154. Lv, Z., et al., *Anticancer peptides prediction with deep representation learning features.* Brief Bioinform, 2021. **22**(5).
155. Agrawal, P., et al., *AntiCP 2.0: an updated model for predicting anticancer peptides.* Brief Bioinform, 2021. **22**(3).
156. Rao, B., et al., *ACPred-Fuse: fusing multi-view information improves the prediction of anticancer peptides.* Brief Bioinform, 2020. **21**(5): p. 1846-1855.
157. Wei, L., et al., *ACPred-FL: a sequence-based predictor using effective feature representation to improve the prediction of anti-cancer peptides.* Bioinformatics, 2018. **34**(23): p. 4007-4016.
158. Rauf, A., et al., *Boosted Prediction of Antihypertensive Peptides Using Deep Learning.* Applied Sciences-Basel, 2021. **11**(5).
159. Manavalan, B., et al., *mAHTPred: a sequence-based meta-predictor for improving the prediction of anti-hypertensive peptides using effective feature representation.* Bioinformatics, 2019. **35**(16): p. 2757-2765.
160. Manavalan, B., et al., *AtbPpred: A Robust Sequence-Based Prediction of Anti-Tubercular Peptides Using Extremely Randomized Trees.* Comput Struct Biotechnol J,

2019. **17**: p. 972-981.
161. Cai, L., et al., *ITP-Pred: an interpretable method for predicting, therapeutic peptides with fused features low-dimension representation.* Brief Bioinform, 2021. **22**(4).
162. Zhang, Y.P. and Q. Zou, *PPTPP: a novel therapeutic peptide prediction method using physicochemical property encoding and adaptive feature representation learning.* Bioinformatics, 2020. **36**(13): p. 3982-3987.
163. Wei, L., et al., *PEPred-Suite: improved and robust prediction of therapeutic peptides using adaptive feature representation learning.* Bioinformatics, 2019. **35**(21): p. 4272-4280.
164. Wei, L., et al., *ATSE: a peptide toxicity predictor by exploiting structural and evolutionary information based on graph neural network and attention mechanism.* Brief Bioinform, 2021. **22**(5).
165. Arif, M., et al., *TargetCPP: accurate prediction of cell-penetrating peptides from optimized multi-scale features using gradient boost decision tree.* J Comput Aided Mol Des, 2020. **34**(8): p. 841-856.
166. Fu, X., et al., *StackCPPred: a stacking and pairwise energy content-based prediction of cell-penetrating peptides and their uptake efficiency.* Bioinformatics, 2020. **36**(10): p. 3028-3034.
167. Qiang, X., et al., *CPPred-FL: a sequence-based predictor for large-scale identification of cell-penetrating peptides by feature representation learning.* Brief Bioinform, 2018.
168. Wei, L., J. Tang, and Q. Zou, *SkipCPP-Pred: an improved and promising sequence-based predictor for predicting cell-penetrating peptides.* BMC Genomics, 2017. **18**(Suppl 7): p. 742.
169. Wei, L., et al., *CPPred-RF: A Sequence-based Predictor for Identifying Cell-Penetrating Peptides and Their Uptake Efficiency.* J Proteome Res, 2017. **16**(5): p. 2044-2053.
170. Hasan, M.M., et al., *HLPpred-Fuse: improved and robust prediction of hemolytic peptide and its activity by fusing multiple feature representation.* Bioinformatics, 2020. **36**(11): p. 3350-3356.
171. Kumar, V., et al., *A Method for Predicting Hemolytic Potency of Chemically Modified Peptides From Its Structure.* Front Pharmacol, 2020. **11**: p. 54.
172. Timmons, P.B. and C.M. Hewage, *HAPPENN is a novel tool for hemolytic activity prediction for therapeutic peptides which employs neural networks.* Sci Rep, 2020. **10**(1): p. 10869.
173. Charoenkwan, P., et al., *iBitter-Fuse: A Novel Sequence-Based Bitter Peptide Predictor by Fusing Multi-View Features.* International journal of molecular sciences, 2021. **22**(16): p. 8958.
174. Charoenkwan, P., et al., *BERT4Bitter: a bidirectional encoder representations from transformers (BERT)-based model for improving the prediction of bitter peptides.*

Bioinformatics, 2021.
175. Charoenkwan, P., et al., *iBitter-SCM: Identification and characterization of bitter peptides using a scoring card method with propensity scores of dipeptides.* Genomics, 2020. **112**(4): p. 2813-2822.
